# MAPK Signaling Determines Anxiety in the Juvenile Mouse Brain but Depression-Like Behavior in Adults

**DOI:** 10.1371/journal.pone.0035035

**Published:** 2012-04-18

**Authors:** Benedikt Wefers, Christiane Hitz, Sabine M. Hölter, Dietrich Trümbach, Jens Hansen, Peter Weber, Benno Pütz, Jan M. Deussing, Martin Hrabé de Angelis, Till Roenneberg, Fang Zheng, Christian Alzheimer, Alcino Silva, Wolfgang Wurst, Ralf Kühn

**Affiliations:** 1 German Research Center for Environmental Health, Institute of Developmental Genetics, Helmholtz Zentrum München, Neuherberg/Munich, Germany; 2 Molecular Neurogenetics, Max-Planck-Institute of Psychiatry, Munich, Germany; 3 Institute of Experimental Genetics, German Research Center for Environmental Health, Helmholtz Zentrum München, GmbH, Neuherberg/Munich, Germany; 4 Institute of Medical Psychology, Ludwig-Maximilians-Universität Munich, Munich, Germany; 5 Institute of Physiology and Pathophysiology, University of Erlangen-Nuremberg, Erlangen, Germany; 6 Department of Neurobiology, University of California Los Angeles, Los Angeles, United States of America; 7 Lehrstuhl für Entwicklungsgenetik, Technical University München-Weihenstephan, Neuherberg/Munich, Germany; 8 Deutsches Zentrum für Neurodegenerative Erkrankungen e.V. (DZNE) Site Munich, München, Germany; Alexander Flemming Biomedical Sciences Research Center, Greece

## Abstract

MAP kinase signaling has been implicated in brain development, long-term memory, and the response to antidepressants. Inducible *Braf* knockout mice, which exhibit protein depletion in principle forebrain neurons, enabled us to unravel a new role of neuronal MAPK signaling for emotional behavior. *Braf* mice that were induced during adulthood showed normal anxiety but increased depression-like behavior, in accordance with pharmacological findings. In contrast, the inducible or constitutive inactivation of *Braf* in the juvenile brain leads to normal depression-like behavior but decreased anxiety in adults. In juvenile, constitutive mutants we found no alteration of GABAergic neurotransmission but reduced neuronal arborization in the dentate gyrus. Analysis of gene expression in the hippocampus revealed nine downregulated MAPK target genes that represent candidates to cause the mutant phenotype.

Our results reveal the differential function of MAPK signaling in juvenile and adult life phases and emphasize the early postnatal period as critical for the determination of anxiety in adults. Moreover, these results validate inducible gene inactivation as a new valuable approach, allowing it to discriminate between gene function in the adult and the developing postnatal brain.

## Introduction

The extracellular signal-regulated kinase/mitogen-activated protein kinase (ERK/MAPK) signaling pathway is a linear kinase cascade with a well-understood biochemistry. Cell surface receptors activate the small GTPase RAS, which itself activates Raf kinases. RAF phosphorylates and activates MEK1 and MEK2 that in turn phosphorylate and activate ERK1 and ERK2 kinases. Finally, activated ERKs either translocate into the nucleus to phosphorylate transcription factors, or directly regulate cytoplasmic substrates. ERK/MAPK signaling is known as a key player in basic cellular processes like growth, differentiation, survival, and apoptosis. Among the three mammalian Raf isoforms, B-RAF is the strongest MEK-ERK activator expressed in neuronal tissues and testis [1] and was shown to be a protooncogene [2]. Furthermore, other members of the ERK/MAPK signaling pathway cause the developmental RAS/MAPK or neuro-cardio-facial-cutaneous (NCFC) syndromes [3], [4].

In the nervous system, the ERK/MAPK signaling pathway is known to participate in long-term potentiation, synaptogenesis, and dendritic differentiation. The global inactivation of B-RAF causes a shutdown of MEK1/2 kinases as exclusive substrates and the resulting depletion of ERK/MAPK signaling leads to embryonic lethality [5]. However, conditional, brain-specific *Braf* knockout mice allow to investigate the role of ERK/MAPK signaling in the nervous system. Thereby it was shown, that *Braf* is essential for long-term potentiation, learning and memory, and synaptic plasticity [6]–[8]. Moreover, ERK/MAPK signaling was linked to neuronal differentiation, axonal growth [9], oligodendrocyte maturation [10], and is associated with autism [11] and the development of depression [12].

In matters of emotional behavior, the acute pharmacological inhibition of the ERK/MAPK signaling using the MEK inhibitor PD184161 leads to depression-like behavior in mice [13]. In addition, activation of ERK/MAPK signaling was found upon treatment with different mood stabilizers like lithium [14], [15] and valproic acid [15], [16]. However, currently little is known about the involved downstream targets of the ERK/MAPK signaling in the brain and about the intracellular cascades and interactions involved in the regulation of anxiety and depression.

To decipher the role of ERK/MAPK signaling for emotional behavior, we studied conditional mouse mutants in which the *Braf* gene becomes inactivated in principle forebrain neurons upon induction in the juvenile or adult brain and compared them to mutants that undergo constitutive inactivation of *Braf* in the juvenile brain. We found that the inactivation of *Braf* in the juvenile or adult brain differentially affects their depression-like behavior or anxiety as adults. Mutants that undergo constitutive inactivation of *Braf* in the juvenile brain were further characterized for alterations of GABAergic neurotransmission, the dendritic complexity of hippocampal neurons, and the global gene expression profile in the hippocampus to identify key targets of neuronal ERK/MAPK signaling. These results reveal a differential function of ERK/MAPK signaling in juvenile and adult life phases and emphasize the early postnatal period as critical for the determination of anxiety in adults.

## Methods

### Animals and treatments

To generate conditional mutants, Braf^flox^ mice (initially derived from R1 ES cells of the 129X1/SvJ×129S1/Sv (“129/Sv") F1 hybrid background) [6] were obtained on a mixed “129/Sv" and FVB genetic background and backcrossed to C57BL/6J mice (obtained from Charles River Germany) for three generations. Braf^icko^ mice were generated by breeding of Braf^flox^ mice with transgenic CamkIIa-CreER^T2^ mice (initially derived on the FVB background) [17], that were backcrossed to C57BL/6J mice for 9 generations. Braf^cko^ mice were generated by breeding of Braf^flox^ mice with transgenic CamkIIa-Cre mice (initially derived on a (CBA/J×C57BL/6J)-F2 background; [18] and R. Kühn, unpublished results) that were backcrossed to C57BL/6J mice for more than 20 generations.

To obtain experimental cohorts, males homozygous for the Braf^flox^ allele were mated with females that were heterozygous for the Braf^flox^ allele and heterozygous for the CamkIIa-Cre or CamkIIa-CreER^T2^ transgene. Littermates homozygous for the Braf^flox^ allele without Cre transgene were used as controls, whereas littermates homozygous for the Braf^flox^ allele and heterozygous for the Cre transgene were used as mutants. This breeding scheme resulted into experimental groups of Braf^cko^ mice with a mean genetic contribution of 93.7% derived from C57BL/6J, of 6.2% from FVB, and of 0.1% from “129/Sv". The experimental groups of Braf^icko^ mice exhibited a similar genetic background derived to 93.4% from C57BL/6J, to 6.5% from FVB, and to 0.1% from “129/Sv". In experiments with Braf^icko^ mice, both mutants and controls were treated with tamoxifen to account for potential non-specific effects of the inducing compound.

Using this breeding scheme, the “flanking allele problem" [19], [20] was avoided for the Braf^flox^ allele, but could not be excluded for the chromosome harboring the heterozygous Cre transgene. To account for a potential phenotypic effect of the CamkII-Cre transgene and its flanking alleles, we compared the behavior of 29 transgenic and 29 non-transgenic CamkII-Cre littermates in the modified holeboard (see Method S2), elevated-plus-maze (EPM), forced swim test (FST), and the accelerating rotarod (see Method S2). This analysis showed that CamkII-Cre transgenic littermates did not exhibit significant differences for the EPM time in open arms, the FST immobility time, the performance on the rotarod, and other parameters (Figure S3). With respect to the CamkIIa-CreER^T2^ transgene we can presently not exclude a potential phenotypic effect of alleles flanking the heterozygous transgene. However, the mutants that were induced at the age of 3–4 weeks or 8–9 weeks exhibit the same genetic background.

Controls and knockouts were group-housed with food and water *ad libitum* in individually ventilated cages under standard laboratory conditions as previously described [21]. Animals were separated based on sex, but not genotype. Induction of Cre in Braf^icko^ mice was achieved by 10 i.p. injections of 40 mg/kg b. wt. tamoxifen (free base, Sigma; solved in 9∶1 sunflower seed oil/ethanol) during five consecutive days. Behavioral testing started at the earliest two weeks after tamoxifen treatment.

### Immunohistochemistry

Mice were euthanised and intracardially perfused with saline, followed by 4% paraformaldehyde in PBS. Brains were prepared, postfixed for 2 h in 4% PFA, and cryoprotected with 25% sucrose in PBS. 40 µm thick, free-floating cryo sections were cut on a microtom and stored in a cryoprotectant. For immunohistochemistry, sections were washed in PBS, bleached with 0.3% H_2_O_2_ in 1∶1 PBS/methanol for 15 min, and blocked with 5% FCS in PBS-T (0.2% Triton-X in PBS) for 30 min at RT. For detection of phosphorylated proteins, TBS was used instead of PBS in all steps. Sections were incubated with rabbit α-BRAF (H-145, 1∶100, Santa Cruz Biotechnology, Heidelberg, Germany) or α-Phospho-p44/42 MAPK (#9101, 1∶50, Cell Signaling Technology, Frankfurt, Germany), respectively, overnight at 4°C, washed with PBS, and incubated with biotin-conjugated α-rabbit antibody (1∶300, Dianova, Hamburg, Germany) for 2 h at RT. For detection, the Vectastain ABC kit (Vector Laboratories, Peterborough, UK) was used and the sections were then mounted, dehydrated, and covered with a resinous mounting medium.

### Western blot analysis

Fresh brain samples were homogenized in RIPA buffer (50 mM Tris-HCl pH 7.4, 1% NP-40, 0.25% sodium deoxycholate, 150 mM NaCl, 1 mM EDTA, protease inhibitor) and protein concentrations were determined by the BCA assay prior to loading on 10% Bis-tris acrylamid gels. After electrophoresis, the proteins were transferred onto PVDF membranes. Next, membranes were blocked with 4% skim milk in TBS-T, incubated with the primary antibodies, washed with TBS-T, and incubated with horseradish-peroxidase-conjugated secondary antibodies (goat α-rabbit, 1∶5 000, Dianova; goat α-mouse, 1∶1 000, Dianova). Signals were visualized with an enhanced chemiluminescence assay (ECL Plus, Amersham, Freiburg, Germany) and quantified with a fluorescence image analyzer. Primary antibodies used were: rabbit α-BRAF, C-19, 1∶600, Santa Cruz Biotechnology; rabbit α-pERK1/2, #9101, 1∶1 000, Cell Signaling Technology; rabbit α-ERK1/2, #9102, 1∶1 000, Cell Signaling Technology; mouse α-βACTIN, AC-15, 1∶100 000, Abcam, Cambridge, UK.

### Behavioral testing

For Braf^icko/juvenile^ behavioral analysis, 26–29 controls and 16–17 mutants were tested in the elevated plus maze and the forced swim test (in this order). For Braf^icko/adult^ behavioral analysis, 29 controls and 29–31 mutants were tested in the elevated plus maze and the forced swim test (in this order). The minimal inter-test interval in all experiments was 6 days. For Braf^cko^ behavioral analysis, 27–29 controls and 22–26 mutants were tested in a first experiment in the light/dark box and the tail suspension test at the age of 16–17 and 24–25 weeks, respectively. A second batch of Braf^cko^ mice, consisting of 24–26 controls and 24–28 mutants, was tested in the elevated plus maze and the forced swim test at the age of 22–23 and 28–29 weeks, respectively. Comparable numbers of male and female animals were used in all experiments.

#### Elevated plus maze

The test arena was made of light grey PVC and consisted of two open arms (30×5×0.3 cm) and two closed arms of the same size with 15 cm walls. The open arms and accordingly the closed arms were facing each other connected via a central square (5×5 cm). The apparatus was elevated 75 cm above the floor by a pole fixed underneath the central square. The illumination level was set at approximately 100 lux in the centre of the maze. For testing, each mouse was placed at the end of a closed arm (distal to the centre) facing the wall and was allowed to explore the maze for 5 min. A camera was mounted above the centre of the maze to video monitor each trial by a trained observer in an adjacent room. The number of entries into each type of arm (placement of all four paws into an arm defining an entry), latency to enter the open arms as well as the time spent in the open and closed arms were recorded by the observer with a hand-held computer. Data were analyzed by using the Observer 4.1 Software (Noldus). After each trial, the test arena was cleaned carefully with a disinfectant.

#### Light/dark box

The test box was made of PVC and divided into two compartments, connected by a small tunnel (4×6×9 cm high). The lit compartment (29×19×24 cm high) was made of white PVC and was illuminated by cold light with an intensity of 650 lux in the middle; the dark compartment (14×19×24 cm high) was made of black PVC and not directly illuminated (approx. 20 lux in the centre). The mouse was placed in the centre of the black compartment and allowed to explore freely the apparatus for 5 min. Behaviors were observed by a trained observer next to the box using a hand-held computer. Data were analyzed with respect to (1) the number of entries, latency to first entry, and time spent in both compartments, and the tunnel; (2) the number of rearings in both compartments and the tunnel. Additionally, grooming behavior was recorded. An entry into a compartment was defined as placement of all four paws into the compartment. Additionally, a camera was mounted above the centre of the test arena to videotape the trial, and the animal's locomotor path in the lit compartment was analyzed with a video-tracking system (Ethovision 2.3, Noldus). The box was cleaned with a disinfectant before each trial.

#### Forced swim test

The forced swimming test was adapted from Ebner *et al.* [22]. The forced swimming apparatus consisted of a cylindrical 10 L glass tank (24.5 cm in diameter) filled to a depth of 20 cm with water (25±1°C). A trained observer recorded the animals behavior in moderate lighting conditions (approx. 30 lux) for 6 min with a hand-held computer scoring the following behaviors: (1) struggling, defined as movements during which the forelimbs broke the water's surface; (2) swimming, defined as movement of the animal induced by movements of the fore and hind limbs without breaking the water surface, and (3) floating, defined as the behavior during which the animal used limb movement just to keep its balance without any movement of the trunk. Data were analyzed by using the Observer 4.1 Software (Noldus). After each trial, the mouse tested was dried with a tissue and put in a new cage, and the water was renewed before testing the next animal.

#### Tail suspension test

For the tail suspension test, each mouse was hung by the base of its tail, fixed with a tape, for 6 min. A trained observer recorded with a hand-held computer the animal's behavior scoring activity and immobility. Data were analyzed by using the Observer 4.1 Software (Noldus).

### Ethics Statement

Experiments on animals were carried out in accordance with the European Community Council Directive of 24 November 1987 (86/609/EEC). All animal experiments were approved by the animal welfare and use committee of the local governmental body (Regierung von Oberbayern, approval numbers 2531-47/03 and -70/07).

### Voluntary wheel running activity

All mice were kept in a 1/23 h light/dark cycle in order to synchronize the circadian activity while preventing the resting phase activity to be affected by light stimuli. 10 control and 10 Braf^cko^ mutant mice (4 females and 6 males each) were housed individually during the experiment in a separate, sound and light insulated room and had access to food and water *ad libitum*. Activity was measured with a Low Profile Wireless Running Wheel (Med Associates Inc., St. Albans, VT, USA) for 17 days and the data was analyzed with the CHRONO software [23].

### Slice electrophysiology

Using standard procedures, transverse hippocampal slices (350 µm thick) were prepared from the brain of male adult Braf^cko^ mice, which were deeply anesthetized with halothane prior to decapitation. The slices were initially maintained in a high-sucrose solution containing (in mM) 75 sucrose, 87 NaCl, 3 KCl, 0.5 CaCl_2_, 7 MgCl_2_, 1.25 NaH_2_PO_4_, 25 NaHCO_3_ and 10 D-glucose. The solution was ice-cold for cutting and warmed to 35°C for 20 minutes immediately after that. The slices were then incubated in modified artificial cerebrospinal fluid (ACSF) containing (in mM) 125 NaCl, 3 KCl, 1 CaCl_2_, 3 MgCl_2_, 1.25 NaH_2_PO_4_, 25 NaHCO_3_ and 10 D-glucose at room temperature (21–24°C) for at least 2 hours before being transferred into the recording chamber individually. Recordings were performed at room temperature in normal ACSF containing (in mM) 125 NaCl, 3 KCl, 2 CaCl_2_, 2 MgCl_2_, 1.25 NaH_2_PO_4_, 25 NaHCO_3_ and 10 D-glucose (pH 7.4), which was exchanged by means of a gravity-driven perfusion system (flow rate 2–3 mL/min). All solutions were constantly gassed with 95% O_2_/5% CO_2_. CA1 pyramidal cells were visualized with a contrast-enhanced CCD camera (Hamamatsu Photonics, Herrsching, Germany). Electrophysiological signals were filtered at 1 kHz (for field potentials) or 2 kHz (for whole-cell currents) and sampled at 10 kHz using a Multiclamp 700B amplifier in conjunction with Digidata 1440A interface and pClamp 10 software (all from Molecular Devices, Sunnyvale, CA, USA). CA1 pyramidal cell population spike (PS) was induced by constant current pulses (pulse width 0.1 ms, every 10 s) delivered to a bipolar tungsten electrode located in CA1 stratum radiatum. The extracellular recording pipette in pyramidal cell layer was filled with modified ACSF, in which bicarbonate was replaced with Hepes to avoid pH change. For whole-cell recording of the evoked inhibitory postsynaptic currents (IPSC) in CA1 pyramidal cells, patch pipettes were filled with solution containing (in mM) 130 CsCl, 3 MgCl_2_, 5 EGTA, 5 Hepes, 2 Na_2_-ATP, 0.3 Na-GTP and 5 QX-314 (pH 7.25). The electrode resistance ranged from 2.5 to 4 MΩ when filled with internal solution. Series resistance in whole-cell configuration was about 10–25 MΩ, which was compensated by 60–80%. Constant current pulses (pulse width 0.1 ms) of 50–200 µA were delivered every 30 s to the concentric bipolar electrode located close to the pyramidal cell layer. IPSCs were recorded at −70 mV, after correcting for liquid junction potentials. GABA_A_ receptor-mediated IPSCs were pharmacologically isolated by perfusing the slices with ionotropic glutamate receptor antagonist kynurenic acid (2 mM). All the drugs were obtained from Sigma. Data were analyzed off-line with Clampfit (Molecular Devices). The PS amplitude was calculated as the averaged value from negative peak to two positive peaks. For evoked IPSCs, we determined peak amplitude, time width at half peak amplitude (T_1/2_) and area above the curve. Spontaneous IPSCs were analyzed using an automated event detection algorithm with an amplitude threshold set as 4 * σ_noise_. The frequency was measured for spontaneous IPSCs.

### Gene expression analysis

Five male Braf^cko^ and six male homozygous Braf^flox^ littermates were killed with CO_2_, the complete hippocampal tissue was prepared, and total RNA was extracted with the Trizol protocol. The integrity and quality of the RNA samples were analyzed with an RNA electrophoresis chip (RNA 6000 Nano Kit, Agilent, Boeblingen, Germany). RNA samples of high integrity and quality (RIN≥7.5) were further processed with the TotalPrep RNA Amplification Kit (Ambion, Austin, TX, USA) and hybridized onto MouseWG-6 v1.1 Expression BeadChips (Illumina, San Diego, CA, USA) following manufacturer's instructions. Data were analyzed using the software R [24] (used packages: beadarray, limma, and vsn). Our microarray data is MIAME compliant and the raw data has been deposited in the NCBI's Gene Expression Omnibus (GEO) database (GEO accession number: GSE33618). For validation of the microarray results, the expression of 22 genes was analyzed by qPCR either with predesigned TaqMan assays (Applied Biosystems, Darmstadt, Germany) or with selfdesigned primer pairs for SYBR Green Assays (Applied Biosystems).

### Neuronal morphology

Two female Braf^cko^ and two female control mice were sacrificed with CO_2_ and brains were prepared and stored in PBS. Golgi impregnation was performed with the FD Rapid GolgiStain Kit (FD NeuroTechnologies, Ellicott City, MD, USA) according to the manufacturer's protocol. Briefly, whole brain hemispheres were impregnated for 14 days at RT in solution A+B, cleared for 3 days in solution C, and then cut with a cryostat in 140 µm coronal slices and mounted onto glass slides. After staining with solution D+E, the slides were dehydrated and covered with a resinous mounting medium. All incubation steps were performed in a dark room. Dendritic morphology was analyzed by manual tracing using the Neurolucida software (MBF Bioscience, Williston, VT, USA). Only granular neurons of the dentate gyrus with a non-truncated dendritic tree and a thorough staining were used for tracing (controls: 138 neurons, mutants: 72 neurons). For analysis of dendritic length and Sholl analysis, the Neuroexplorer software (MBF Bioscience) was used.

### Statistical analysis

All data are reported as the means ± S.E.M. unless otherwise stated. Statistical comparisons were performed by Student's t-test or by analysis of variance (ANOVA) with the SPSS software (SPSS Inc., Chicago, IL, USA). The accepted level of significance was *P*<0.05. For statistical analysis of behavioral data, we performed first a two-way ANOVA with sex as independent variable to check for sex×genotype interactions. Since no interactions were found, data from male and female mice were pooled and Student's t-tests or one-way ANOVAs were performed on the pooled samples. For the sake of clarity, only pooled data are presented in the figures and reported in the results section.

As observations of neuronal morphology were made in cells with several cells from each of 2 wildtypes and 2 mutants, linear mixed-effects models with animal as random effect variable were applied to perform group comparisons and calculate confidence intervals. For all calculations, the R software [24] with the nlme package [25] was used.

## Results

### Generation of inducible *Braf* knockout (Braf^icko^) mice

The characterization of ERK/MAPK function specifically in the juvenile or the adult brain requires a timed control of gene inactivation. For this purpose, and since the complete loss of *Braf* leads to embryonic lethality [5], we used a tamoxifen-inducible CamkIIa-CreER^T2^ mouse line that exhibits inducible Cre activity in principal neurons of the cortex, hippocampus, medial striatum, and hypothalamus [17]. By crossing CamkIIa-CreER^T2^ with Braf^flox^ mice [6], we generated inducible conditional *Braf* knockout (“Braf^icko^") mice, in which the inactivation of *Braf* is restricted to neurons that express Cre recombinase and delete exon 14 of the Braf^flox^ allele (Figure S1A). As both, control and mutant Braf^icko^ mice are homozygous for the loxP flanked *Braf* allele, these groups do not differ with respect to alleles flanking the targeted locus [19], [26]. Using a Cre reporter strain, we confirmed the reported recombination pattern of CamkIIa-CreER^T2^ mice [17] and further found that recombination can be induced in the brain of juvenile (3-weeks-old), adolescent (6-weeks-old), and adult mice (14-weeks-old) at equal efficiency (Figure S1B).

### Emotional behavior of adult and juvenile Braf^icko^ mice

**Figure 1:**
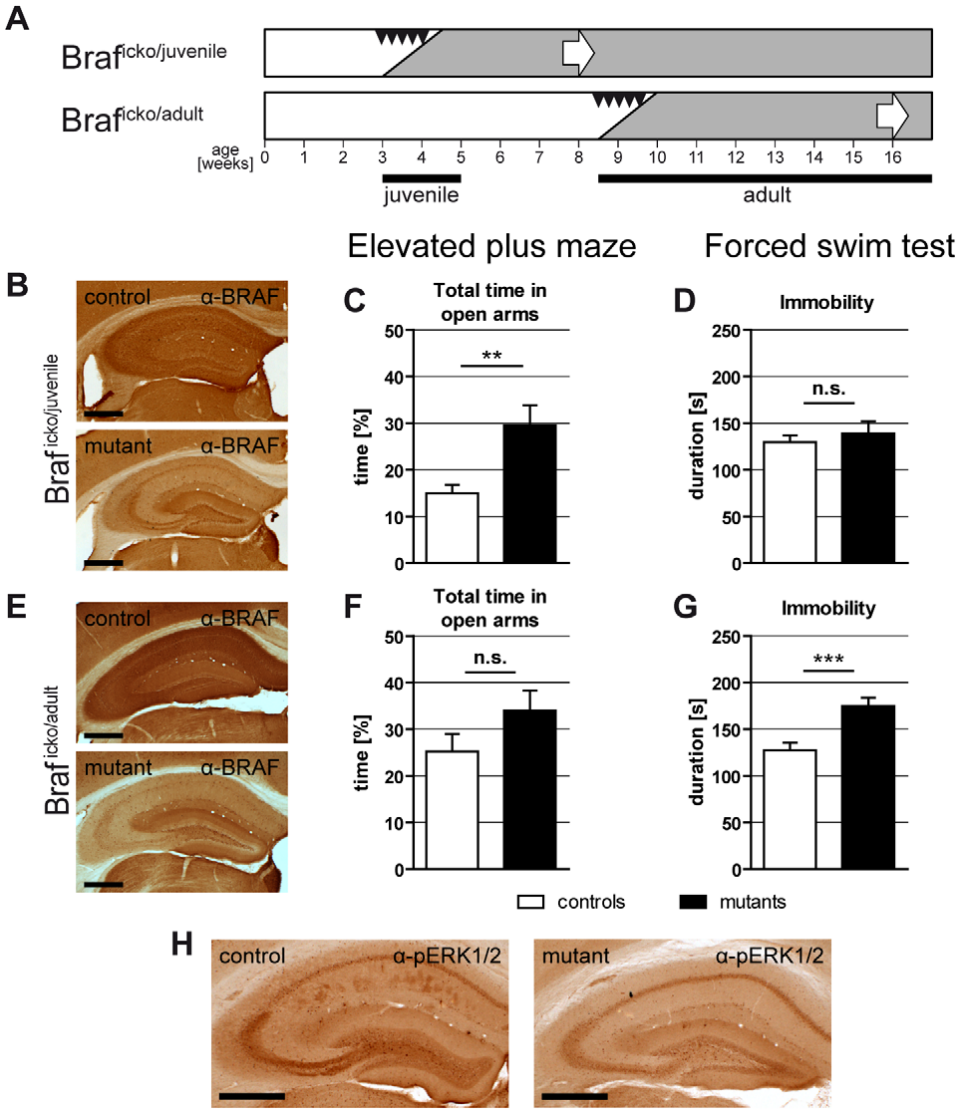
Comparison of emotional behaviors between early and late induced Braf^icko^ mice. (**A**) Scheme of experimental procedure: Juvenile and early adult Braf^icko^ mice were induced with tamoxifen at an age of 3–4 and 8.5–9.5 weeks, respectively (black arrowheads) and behavioral analysis started at week 8 and 16, respectively (white arrows). (**B,E**) Immunohistochemical detection of BRAF of juvenile (**B**) and early adult (**E**) Braf^icko^ mice revealed depletion of the protein in principle neurons of the hippocampus (shown) and the cortex (not shown). Scale bars represent 500 µm. (**C,F**) In the elevated plus maze, a less anxious behavior was observed in Braf^icko/juvenile^ (**C**), but not in Braf^icko/adult^ mice (**F**). (**D,G**) In the forced swim test, Braf^icko/adult^ mice displayed an increase in depression-like behavior (**G**) that was not present in juvenile induced Braf^icko^ mice (**D**) (n.s.: not significant, **: *P*<0.01, ***: *P*<0.001). (**H**) Immunohistochemical detection of Phospho-ERK1/2 in Braf^icko/juvenile^ mice demonstrated a strong reduction of ERK/MAPK signaling in principle neurons of the hippocampus of Braf^icko^ mutants. Scale bars represent 500 µm.

Adult or juvenile Braf^flox/flox;CreERT2^ and Braf^flox/flox^ mice were both treated for 5 days with tamoxifen and classified as Braf^icko^ mutants and treated controls, respectively. Braf^icko^ mice were first induced at 8.5 weeks (“Braf^icko/adult^ mutants and controls") to test for ERK/MAPK function specifically in the adult forebrain, excluding postnatal developmental effects (Figure 1A). Four weeks after the completion of induction Braf^icko/adult^ mice were analyzed for anxiety-related and depression-like behavior in the elevated plus maze (EPM) and the forced swim test (FST) respectively, followed by the confirmation of BRAF depletion in brain sections (Figure 1E). Interestingly, in the FST the Braf^icko/adult^ mutants showed significantly increased passive behavior (Figure 1G; *t* [_58_] = 3.943; *P*<0.001) whereas in the EPM no differences compared to controls were found (Figure 1F). These results indicate an increase of depression-like behavior and normal anxiety-like behavior in Braf^icko/adult^ mutants. Next, Braf^icko^ mice were induced early postnatal at 3 weeks of age (“Braf^icko/juvenile^ mutants and controls") to cause juvenile inactivation of ERK/MAPK signaling in forebrain neurons (Figure 1A). At the age of 8 weeks Braf^icko/juvenile^ mice were analyzed for anxiety-related and depression-like behavior in the EPM and FST. Upon completion of the phenotype analysis, the depletion of the BRAF protein in forebrain regions of Braf^icko/juvenile^ mutants was confirmed by immunohistochemistry (Figure 1B). Braf^icko/juvenile^ mutants spent significantly more time in the open arms of the EPM (Figure 1C; *t* [_40_] = 3.513; *P*<0.01), whereas no difference in the passive behavior in the FST was observed (Figure 1D). These results indicate a normal depression-like behavior but decreased anxiety of adults upon *Braf* depletion in the juvenile brain. Taken together, these results suggest a differential role of ERK/MAPK signaling for emotional behavior in the juvenile *versus* the adult brain. In agreement with previous results from pharmacological intervention, the inhibition of ERK/MAPK signaling in the adult brain causes increased depression-like behavior. In contrast, the early deficiency of ERK/MAPK activity in the postnatal brain between 4 and 8 weeks leads to strongly decreased anxiety, but unaltered depression-like behavior of adults. Therefore, neuronal ERK/MAPK signaling is essential to establish normal anxiety- or depression-like behavior, in dependence on the timing of gene inactivation. Since in the juvenile brain, neuronal ERK/MAPK signaling is a critical component to establish normal anxiety, we assume that an early ERK/MAPK deficiency causes long lasting alterations in brain development.

### Generation of constitutive *Braf* knockout (Braf^cko^) mice

**Figure 2:**
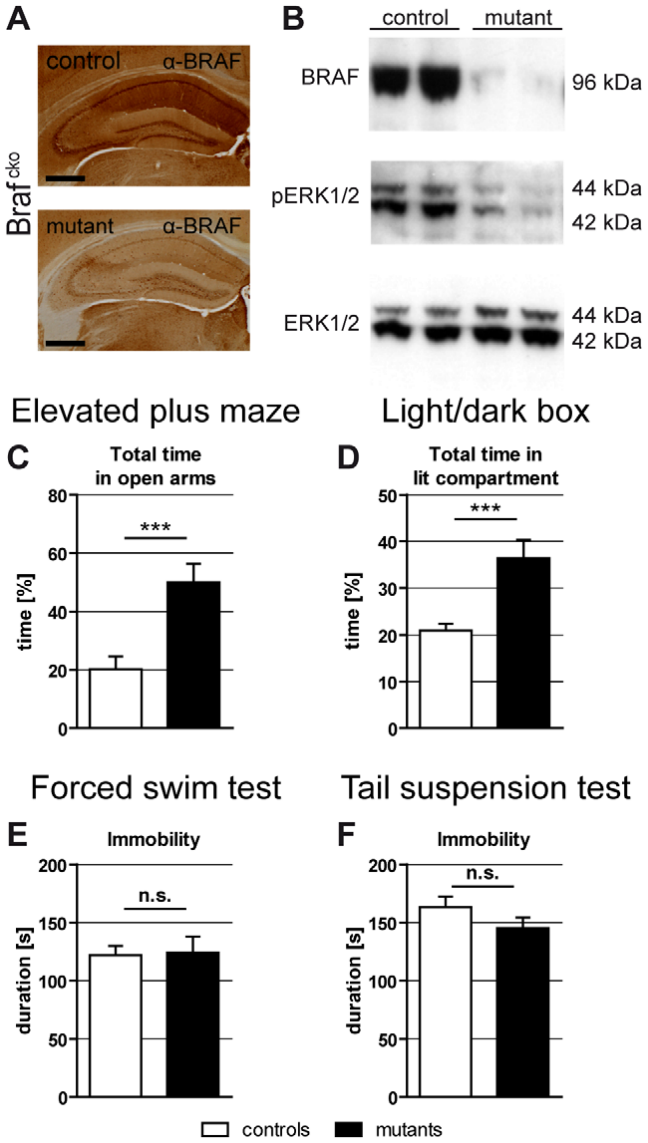
Molecular analysis and emotional behavior of Braf^cko^ mice. (**A**) Immunohistochemical detection of BRAF of Braf^cko^ mice revealed depletion of the protein in the hippocampus (shown) and the cortex (not shown). (**B**) Western blot analysis of hippocampal samples of Braf^cko^ mice validates the depletion of BRAF protein and the subsequent inactivation of ERK/MAPK signaling. While total levels of ERK1/2 in Braf^cko^ mice were unaffected, a strong reduction, but no complete depletion of Phospho-ERK1 and -ERK2 was observed in the hippocampus of mutants. The remaining ERK1/2 activity in Braf^cko^ forebrain regions likely results from non-glutamatergic neuronal or glial cells that express BRAF, but which do not express the CamkIIa-Cre transgene (see Figure S2I). (**C**) Elevated plus maze: Mutant mice spent significantly more time in the open arms of the maze. (**D**) Light/dark box: *Braf* mutants spent more time in the aversive lit compartment. (**E,F**) No changes in depression-like behavior were observed in Braf^cko^ mutants in the forced swim test (**E**) and the tail suspension test (**F**). (n.s.: not significant, ***: *P*<0.001).

To further analyze developmental alterations in the juvenile brain we continued our studies using forebrain-specific, early postnatal conditional Braf mutants which render the stressful application of tamoxifen for inducing the knockout unnecessary. These conditional *Braf* mutants (“Braf^cko^") were generated by the cross of Braf^flox^ mice with the CamkIIa-Cre line [18]. In Braf^cko^ mice, inactivation of the *Braf* gene in principal forebrain neurons begins 2 weeks after birth and is complete at the age of 6 weeks (as shown by Southern blot analysis, Figure S2B) leading to a strong reduction or complete abolishment of ERK/MAPK signaling in affected neurons (Figure 2B), which recapitulates the spatial and temporal inactivation of *Braf* in Braf^icko/juvenile^ mutants (Figure 2A and S2). To rule out possible effects of the Cre transgene on behavior, CamkIIa-Cre mice (backcrossed >20 generations to the C57BL/6J background) were compared to non-transgenic littermates for anxiety- and depression-related parameters. This analysis showed that the CamkII-Cre transgene does not cause significant differences in behavior compared to wildtype littermate controls (Figure S3).

### Emotional behavior and activity pattern of Braf^cko^ mice

The anxiety-related behavior of Braf^cko^ mice was assessed in the EPM and light/dark box (LD). In both tests we found that Braf^cko^ mice spent significantly more time in the aversive compartments than the controls (EPM open arms, Figure 2C; *t* [_47_] = 3.912; *P*<0.001) (LD lit compartment, Figure 2D; *t* [_53_] = 3.889; *P*<0.001), suggesting a reduced anxiety-related behavior of *Braf* deficient mice. Other behavioral parameters like the total distance travelled, the mean velocity, and the number of entries in the EPM and LD zones were not different between the groups (data not shown). In both paradigms for behavioral despair, Braf^cko^ mutants showed no differences in their depression-like behavior. In the FST (Figure 2E), as well as in the tail suspension test (TST, Figure 2F), the total duration of passive behavior (immobility) was unchanged between mutant and control animals, indicating no effect of the *Braf* knockout on depression-like behavior. In conclusion, Braf^cko^ mutants showed, like Braf^icko/juvenile^ mutants, reduced anxiety-like behavior but normal depression-like behavior. Notably, the behavioral changes due to the juvenile depletion of ERK/MAPK signaling are independent of the time point of behavioral testing (early adult in Braf^icko/juvenile^ and late adult in Braf^cko^).

**Figure 3:**
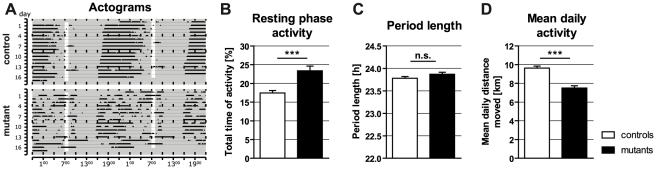
Daily voluntary wheel running activity in *Braf* mutants and controls. (**A**) Representative actograms of Braf^cko^ and control mice. The Braf^cko^ mouse (lower panel) showed a much more fragmented pattern in a LD 1∶23 experiment compared to its floxed littermate (upper panel). Vertical white bar represents daily light phase. (**B**) Activity during resting phase is significantly increased in Braf^cko^ mice. (**C**) Period length analysis revealed no differences between floxed (23.78±0.04) and Braf^cko^ mice (23.87±0.04). (**D**) Mean daily voluntary wheel running activity over 17 days was decreased in Braf^cko^ mice (7.51±0.23) compared to floxed (9.63±0.22). (n.s.: not significant, ***: *P*<0.001).

ERK/MAPK signaling has been proposed as regulator of circadian rhythms in the suprachiasmatic nucleus [27], [28]. To examine whether Braf^cko^ mice exhibit an altered circadian rhythm, we measured their voluntary wheel running activity in a 1/23 h light/dark experiment for 17 days. As shown for two representative mice in Figure 3A, there was no general shift of time of activity between mutants and controls. Both genotypes synchronized the beginning of their activity phase to ∼4 pm. Interestingly, mutant mice showed a much more fragmented activity pattern and a less distinct end point of their activity. Moreover, Braf^cko^ mice showed a significantly increased activity during the resting phase compared to the floxed controls (Figure 3B; *t*_[225]_ = 4.266; *P*<0.001). Despite these different patterns, the overall diurnal period length was not changed in the Braf^cko^ mice (Figure 3C). In contrast, the mean daily activity, measured by the total distance moved per day, was significantly reduced in the Braf cKOs (Figure 3D; *t*_[329]_ = 6.772; *P*<0.001).

### Analysis of GABAergic signaling of Braf^cko^ mice

**Figure 4:**
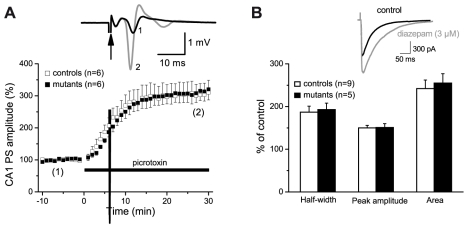
GABA_A_ receptor-mediated inhibition is not enhanced in hippocampi of Braf^cko^ mice. (**A**) Extracellular recording of population spikes in CA1 pyramidal cell layer evoked by electrical stimulation of the Schaffer collateral/commissural pathway. Peak amplitudes of population spikes were normalized before application of GABA_A_ receptor antagonist, picrotoxin (100 µM). Inset depicts individual population spikes recorded at like-numbered time points of the graph in a control slice. Arrow indicates electrical stimulation of afferent pathway. (**B**) Whole-cell recording of IPSCs from CA1 pyramidal cells. Application of diazepam (3 µM) enhanced IPSC amplitude and slowed decay (inset). Effects of diazepam were quantified with respect to half width, peak amplitude, and area of IPSC. Histograms did not reveal any differences between genotypes.

GABAergic neurotransmission is intimately involved in the regulation of emotional behavior and GABA_A_ receptors are well-established targets of anxiolytic drugs. Furthermore, a direct regulation of GABA_A_ receptors by ERK mediated phosphorylation has been demonstrated [29]. Therefore, increased GABAergic neurotransmission may contribute to the altered emotional phenotype of Braf^cko^ mice. In a first set of experiments, we performed extracellular recordings of population spikes of CA1 pyramidal cells in hippocampal slices of Braf^cko^ and control mice. Determination of input-output relationships, in which population spike amplitudes were plotted as function of increasing stimulus strength, demonstrated proper functioning of the Schaffer-CA1 synapse in hippocampi from both genotypes under conditions of low-frequency stimulation (Figure S4A). Pharmacologic suppression of GABA_A_ receptor-mediated inhibition by the GABA_A_ receptor antagonist picrotoxin produced a strong enhancement of the population spike amplitude that was virtually indistinguishable between hippocampi from the two genotypes (Figure 4A). We next performed whole-cell recordings from voltage-clamped CA1 pyramidal cells to directly examine and compare pharmacologically isolated inhibitory postsynaptic currents (IPSCs) in the two preparations. In none of these recordings, we obtained evidence for enhanced GABAergic neurotransmission in mutant hippocampi (Figure S4B–D). Furthermore, IPSCs from CA1 pyramidal cells of Braf^cko^ mice proved as sensitive to the augmenting effect of diazepam as those from controls (Figure 4B). These results argue against the notion that up-regulation of GABA_A_ receptor-mediated inhibition underlies or appreciably contributes to the altered emotional behavior of *Braf^cko^* mutants.

### Neuronal arborization in the hippocampus of Braf^cko^ mice

**Figure 5:**
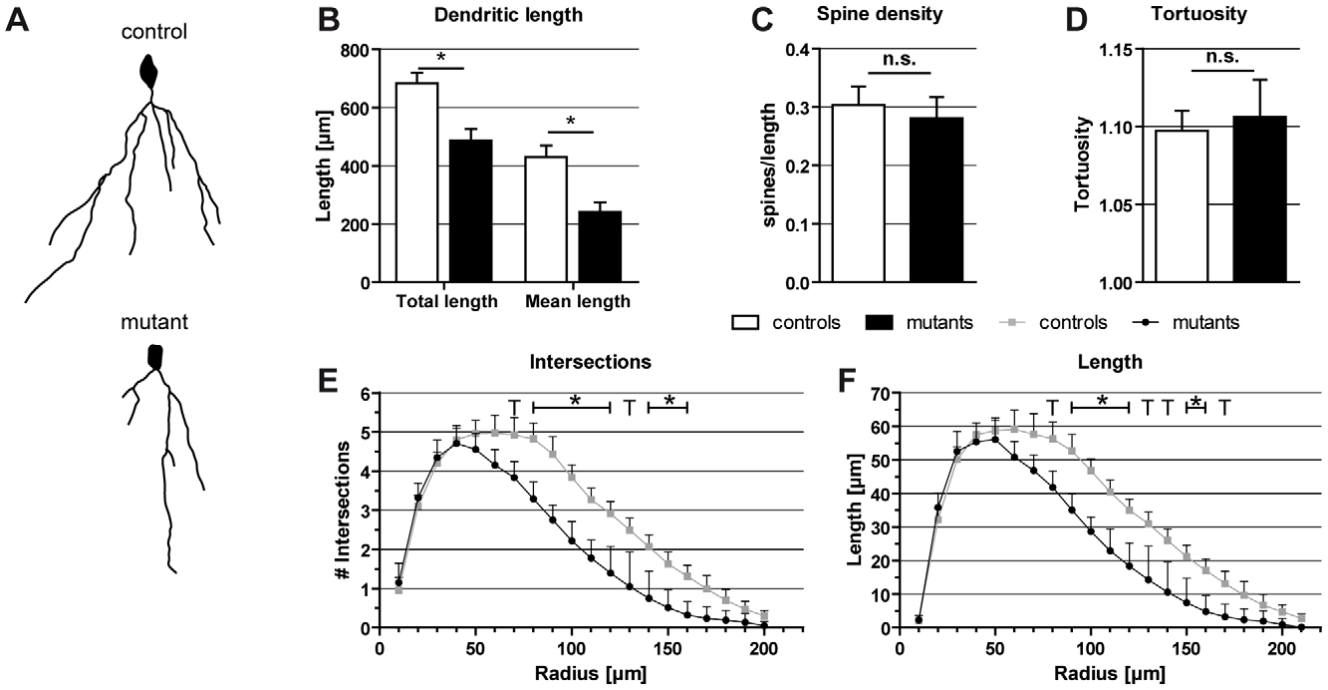
Neuronal morphology of granular neurons in dentate gyrus of Braf^cko^ mice. (**A**) Camera lucida drawings of representative granule neurons of Braf^cko^ controls and mutants. (**B**) Total and mean length of dendrites was significantly reduced in granular neurons of *Braf* deficient mice. (**C,D**) Spine density and tortuosity were not altered due to *Braf* knockout. (error bars: 95% confidence interval; n.s.: not significant, *: *P*<0.05) (**E,F**) Sholl analysis of the dendritic branching revealed an overall reduction of complexity of the granular neurons. Number of intersections (**E**) and dendritic length (**F**) were significantly reduced in the distance of 80–120 and 140–160 µm (intersections) and 90–120 and 150–160 µm (dendritic length) from soma, respectively (T: tendency, *: *P*<0.05).

Alterations in emotional behavior are often associated with changes in dendritogenesis and spine density [30]–[33]. Since the microarray analysis of *Braf* mutants (see below) identified differentially expressed genes related to neuronal growth, development, and arborization (Table S2), we performed Golgi stainings of dentate gyrus granular neurons of Braf^cko^ mice. This analysis revealed that the total and the mean length of dendrites were significantly reduced in these cells (Figure 5B; total length: *P*<0.05, mean length: *P*<0.05). Sholl analysis of the tracing data showed that in *Braf* mutants both the number of intersections (Figure 5E) and the interradial length of the dendritic arbores (Figure 5F) are strongly decreased in the distance of 80–160 µm (intersections) and 90–160 µm (interradial length) from the soma, respectively. These results demonstrate an overall reduction of the complexity of granular neurons by an impaired arborization in the *Braf* mutants. In contrast, the spine density and tortuosity of dentate gyrus granular neurons were not significantly different between *Braf* mutants and controls (Figure 5C and D), indicating that ERK/MAPK signaling does not control spine formation and dendritic routing.

### Global gene expression analysis in the hippocampus of Braf^cko^ mice

A major molecular function of ERK/MAPK signaling is the regulation of gene expression by the activation of transcription factors. To identify genes that are controlled by ERK/MAPK signaling in principle forebrain neurons, we compared the global gene expression in the hippocampus of adult Braf^cko^ and Braf^flox^ control males. This analysis revealed 111 genes that were downregulated (*P_adj_*<0.05) and 39 genes that were upregulated (*P_adj_*<0.05) in Braf^cko^ mice (Table S1). To control for the impact of the Cre transgene on gene expression, we compared the hippocampal gene expression of CamkIIa-Cre and wildtype littermates. This analysis revealed four differentially expressed transcripts (*3110047M12Rik*, *Oxr1* [2 transcripts], *Zfpm2*; Table S1) that were excluded from the further study. For validation of the microarray data by qPCR, we selected 22 transcripts representing transcription factors (e.g. *Egr1*, *Egr4*, *Etv5*), immediate early genes (e.g. *Bdnf*, *Egr1*, *Egr4*), genes related to behavior (e.g. *Npy*, *Bdnf*, *Cck*), to neuronal development (e.g. *Bdnf*, *Klk8*, *Etv1*), and to circadian rhythm (*Per2*, *Bdnf*, *Cck*). Using biological replicates from mutants and controls of the same sex and age this reanalysis confirmed the microarray results in all 22 cases (Table S2).

To further identify genes that are directly controlled by ERK/MAPK signaling, the promoter regions of all 111 differentially downregulated genes were analyzed *in silico* for binding sites of the two major ERK-dependent transcription factors CREB1 and ELK1. Furthermore, we analyzed the evolutionary conservation of these motifs, assuming that a wide conservation indicates the central function of a gene in neuronal ERK/MAPK signaling [34]. We identified nine genes with at least one binding site for CREB1 or ELK1 that were conserved between two or more species (Table 1; for more detailed results see Table S3). The promoters of four of these genes show sites for both transcription factors, four genes harbor only CREB1 sites and one gene contains only an ELK1 site. Interestingly, highly conserved CREB1 sites (≥4 orthologs) were found in the promoters of the somatostatin (*Sst*), glutamate receptor 3 (*Gria3*), dual specificity phosphatase 4 (*Dusp4*), early growth response 1 (*Egr1*), *D15Wsu169e*, and brain derived neurotrophic factor transcript variant 3 (*Bdnf*) genes of up to eight mammalian species. Highly conserved ELK1 sites were identified in the promoters of *Dusp4*, *Egr1*, zinc finger protein 326 (*Zfp326*), and dual specificity phosphatase 5 (*Dusp5*) (Table 1 and Table S3). The expression levels of six of these genes was reanalyzed by qPCR and revealed the reduction of transcripts in the hippocampus of Braf^cko^ mice by 12%–78% (Table S2).

**Table 1:** Bioinformatic prediction of CREB1 and ETS/SRF target genes.

Gene symbol	microarray (fold change)	qPCR (fold change)	Binding site (BS)	# of species	Conserved BS per Module
*Sst*	−1.80	n.t.	CREB1	10	2
*Gria3*	−1.34	−1.14	CREB1	8	1
*Dusp4*	−1.86	−2.86	CREB1	8	1
			ETS/SRF	7	1
*Egr1*	−2.13	−3.57	CREB1	6	2
			ETS/SRF	4	4
*D15Wsu169e*	−1.29	n.t.	CREB1	6	1
			ETS/SRF	2	1
*Zfp326*	−1.36	n.t.	ETS	5	1
*Bdnf (transcript variant 3)*	−1.41	−2.27	CREB1	4	1
*Dusp5*	−1.90	−3.33	CREB1	3	1
			ETS/SRF	4	1
*Egr4*	−2.24	−2.94	CREB1	3	2

Differentially downregulated genes from Braf^cko^ mice were analyzed for the presence of evolutionary conserved CREB1 and ETS/SRF target sites. Nine genes were found representing putative functional ERK/MAPK pathway effectors (for more detailed results, see Table S3). (n.t.: not tested).

While *Dusp4*, *Dusp5*, *Egr1*, and *Egr4* are known targets of ERK-dependent transcription in the CNS, the other targets are newly described. Interestingly, *Sst*, *Gria3*, *Egr1*, and *Bdnf* have been previously linked to emotional behavior as well as to LTP and memory, indicating a connection of these processes by MAPK stimulated gene expression.

## Discussion

ERK/MAP kinase signaling has been implicated in brain development, long-term memory, and in the response to antidepressants. Conditional *Braf* knockout mice enabled us to unravel a new role of ERK/MAPK signaling for emotional behavior. Braf^icko/juvenile^ and Braf^cko^ mutants, that complete *Braf* inactivation in principle forebrain neurons by the age of 4 or 6 weeks respectively, exhibited decreased anxiety-like behavior in the LD and EPM. We focused the further analysis of the mutant's phenotype on the hippocampus since this region has been shown to play an important role in emotional processing [35]–[37].

Since inhibitory GABAergic neurotransmission is a key modifier of anxiety, the anxiolytic phenotype of Braf^cko^ mice might have been caused by an increased release of GABA or an enhanced response of GABA_A_ receptors. A direct negative regulation of GABA_A_ receptors by ERK mediated phosphorylation has been demonstrated *in vitro* for the α1 subunit [29]. However, the analysis of GABAergic neurotransmission revealed the normal function of this system in the hippocampus of Braf^cko^ mice and does not explain their anxiolytic phenotype. A major function of ERK/MAPK signaling is the regulation of gene expression by the activation of the transcription factors CREB1 and ELK1. Gene expression analysis in the hippocampus of Braf^cko^ mutants revealed numerous up- or downregulated transcripts that could account for the behavioral and morphological phenotypes of these mutants. To identify the direct transcriptional targets of neuronal ERK/MAPK signaling, we analyzed the promoter regions of all differentially downregulated transcripts for the presence of CREB1 and ELK1 binding motifs. This analysis revealed an evolutionary conserved group of nine transcripts that can be regarded as the core of ERK/MAPK responsive genes in hippocampal neurons. Besides the immediate early genes *Egr1*/*Egr4* and the regulatory phosphatases *Dusp4*/*Dusp6*, which are known targets of MAPK signaling, we identified five genes that were previously unrecognized as ERK/MAPK targets. Three of these genes, early growth response 1 (*Egr1*), somatostatin (*Sst*), and brain-derived neurotrophic factor (*Bdnf*, transcript 3), are of particular interest since they play a role in neuronal activity and behavioral modification. Mutant mice with a complete depletion of the transcription factor *Egr1* show a strong anxiolytic phenotype in the elevated plus maze [38]. In *Braf* mutants, that exhibit reduced levels of *Egr1* mRNA, a similar effect was found in the two anxiety-related behavior tests. The somatostatin system subserves neuromodulatory roles in the brain, influencing motor activity, sleep, sensory processes, and cognitive functions. *Sst* expression has been found altered in bipolar disorder [39] and shown to control the migration of developing neurons [40]. A deficiency of mature BDNF and its receptor TrkB has been implicated in increased anxiety-related behavior and decreased neuronal complexity of hippocampal neurons [31], [41]. In Braf^cko^ mice, we observed a reduction of the *Bdnf* transcript variant 3 that initiates at the *Bdnf* promoter IV. This promoter was shown to be an initial target in the therapeutic action of the mood stabilizers lithium and valproic acid [42]. Furthermore, ERK/MAPK signaling has been found *in vitro* to act as positive regulator of dendritogenesis [43] and reduced neuronal complexity is also found in *Bdnf* and *TrkB* mutants. These mutants, however, exhibit increased anxiety behavior [31], [41]. In future studies it would be therefore interesting to further study the role of BDNF in mediating emotional behavior in *Braf* mutants and to investigate whether *Braf* mutants are still sensitive to antidepressant treatment or whether the loss of ERK/MAPK signaling and subsequent *Bdnf* inactivation prevent their action.

Finally, Mozhui et al. [44] found a correlation between stress-induced alterations in anxiety-related behavior and the Grin2a NMDA receptor, a known upstream activator of the ERK/MAPK pathway, which was accompanied by an overlap in gene expression alterations.

It is therefore possible that the reduced expression of these “core" genes and their downstream targets cause the altered anxiety behavior, but also the reduced neuronal complexity and the fragmented activity during the sleep period of Braf^cko^ mice. However, at present we cannot attribute the action of individual regulated genes directly to specific phenotypes of Braf^cko^ mice. As shown for *C. elegans*, ERK/MAPK controlled biological phenotypes are mediated by functional groups of 2–10 interacting proteins [45].

The fragmented activity pattern of Braf^cko^ mice and their increased activity during the resting phase may represent a disturbance of sleep, e.g. a delayed sleep onset. Since the disruption of sleep and circadian activity are associated with psychiatric disorders [46] it will be interesting to further study the sleeping behavior of *Braf* mutants and to investigate in inducible Braf^icko^ mice whether the fragmented activity phenotype results from developmental effects. Despite the alterations in gene expression, neuronal morphology, and reduced anxiety, Braf^cko^ mice exhibited normal depression-like behavior as measured by the FST and TST paradigms that are used to assess behavioral despair [47].

The influences of developmental gene knockouts on anxiety-related behavior have been widely discussed [48], and the early postnatal period has been found critical for the development of neural circuits that mediate anxiety and suggest the neurodevelopmental origin of anxiety disorders [49]. Using inducible 5-HT_1A_R deficient mice, it has been shown that adults exhibit increased anxiety if 5-HT_1A_R is absent during the third and fourth postnatal week, but not if it is absent in the adult brain [50]. During the postnatal period, the hippocampal neurocircuitry is rapidly maturing. For example, the development of basal and apical dendrites is achieved during the first to fourth week of life [51]. In addition, the formation of axonal terminals and dendritic spines in the hippocampal CA1 region is not completed before day 24 [52]. Since *Braf* becomes inactivated in Braf^cko^ mice 2–6 weeks after birth, we asked whether the anxiolytic phenotype of these mutants originates from postnatal developmental alterations. We compared inducible mutants that underwent *Braf* inactivation either in the early postnatal development or the adult brain. We found that the anxiolytic phenotype occurred only in *Braf* mutants that were induced at 3 weeks but not at the age of 9 weeks. This result suggests that a juvenile ERK/MAPK deficiency in forebrain principle neurons leads to long-lasting neurodevelopmental alterations that cause reduced anxiety in adults, providing further evidence for the postnatal period as critical for the establishment of normal anxiety behavior. As found for 5-HT_1A_R [50], in the adult brain ERK/MAPK signaling is not directly linked to anxiety. Therefore, it will be of interest to further study whether the anxiolytic phenotype of juvenile *Braf* mutants may be linked to alterations in serotonergic neurotransmission. In contrast to Braf^icko/juvenile^ and Braf^cko^ mice, Braf^icko/adult^ mutants exhibited reduced activity in the FST, suggesting that in the adult brain, ERK/MAPK signaling plays a role in the modulation of depression-like behavior. This finding is consistent with previous observations that associated ERK/MAPK signaling with depression-like behavior in adult mice and rats. Treatment of mice with the MEK inhibitor PD184161 increases depression-like behavior [13] whereas an increase of ERK/MAPK signaling was found upon treatment with the mood stabilizers lithium [14], [15] and valproic acid [15], [16]. In addition, it has been recently described that depressive subjects exhibit an increased activity of the MAPK phosphatase MKP-1 and that MKP-1 overactivity causes depressive behaviors in rodents [53]. Our results confirm the direct relationship of ERK/MAPK signaling and depression-like behavior but restrict this relation to the adult brain. The functional redirection of ERK/MAPK signaling may be mediated by distinct sets of activator and effector molecules that transduce different signals in the juvenile *versus* the adult brain. The ERK/MAPK pathway is known to be activated during embryogenesis by fibroblast growth factor receptors (FGFR) [54], while in the adult brain CRH receptor 1 and glucocorticoid receptor were shown to activate ERK [55], [56]. All of these receptors have been implicated in the modification of anxiety- and depression-like behavior, but further studies are required to define which of these receptors, or other receptors, are coupled to the ERK/MAPK pathway in the juvenile brain.

Our study is the first reporting a role of neuronal ERK/MAPK signaling for anxiety-like behavior in juvenile brain development and for depression-like behavior in the adult brain. The latter finding confirms that ERK/MAPK signaling is essential for the expression of normal behavior in the FST. Therefore, the MAP kinase phosphatases MKP-2, -3, -4, or -6, which mediate negative feedback inhibition of ERK kinases [57], may represent new targets for the development of antidepressive drugs. Given the effect of ERK/MAPK on emotional behavior, the genes that constitute this pathway could also play a role in the etiology of mental disorders. Our results suggest that alleles for *Braf* and other members of the ERK/MAPK pathway may represent risk factors for the development of psychiatric diseases, either by copy number variations in affected genotypes [58] or by altered epigenetic control of gene expression.

Besides the Braf^cko^ and Braf^icko/juvenile^ mutants, various knockout mouse models exhibit reduced adult anxiety-like behavior, like conditional mutants for *Crhr1* or the glucocorticoid receptor *Nr3c1* gene [59], [60]. Since gene inactivation in these models occurs before or during juvenile brain development by the use of noninducible Cre driver lines, it is possible that phenotypes reported from such early postnatal mutants represent developmental alterations and do not report the functional role of these genes in the adult brain.

Our results provide the first demonstration that an inducible, Cre/loxP-based conditional mutant allows to differentiate between gene function in the juvenile and adult brain. The future application of inducible gene inactivation in the brain is greatly facilitated by the International Knockout Mouse Consortium (IKMC, www.knockoutmouse.org) that provides Cre/loxP conditional alleles in ES cells and mice on a genome-wide scale. Moreover, since knockout mice are also used for the validation of drug targets [61], inducible gene inactivation could allow a better prediction of the action of future drugs in the adult brain. Our results from Braf^icko^ mice show that inducible gene inactivation faithfully reports on gene function in the adult brain and provides a valuable tool to study the genetic basis of psychiatric diseases.

## Supporting Information

Figure S1 **Structure of the Braf^flox^ allele and expression pattern analysis of the CamkIIa-CreER^T2^ mouse.** (**A**) In the Braf^flox^ allele, exon 14 of *Braf* is flanked by two Cre recombinase recognition (loxP) sites. Cre mediated excision of this exon leads to a reading frame shift and the production of a truncated, non-functional BRAF protein. (**B**) CamkIIa-CreER^T2^ mice were bred with the Gt(ROSA)26Sor Cre reporter line that exhibits β-galactosidase activity upon Cre recombination. Strong recombinase activity was observed in the cortex and hippocampus. Medium activity was found in striatum and hypothalamus. No differences for the induction ability of the CreER^T2^ were found between the 3^rd^, 6^th^, and 14^th^ week of age.
(PDF)

Figure S2 **Molecular characterization of CamkIIa-Cre and Braf^cko^ mice.** (**A**) Using a Cre reporter strain (Soriano, 1999), we confirmed the activity of the CamkIIa-Cre transgene in principle forebrain neurons (olfactory bulb (oB), cortex (Cx), hippocampus (Hc), and striatum (St)) and further observed recombination in the hypothalamus (Ht) and inferior colliculus (Ic), but not in the thalamus (Th), hindbrain (Hb), brainstem (Bs), or cerebellum (Cb). (**B**) Using Southern blot analysis of genomic DNA (Method S1), we found that the inactivation of *Braf* in the hippocampus and cortex (data not shown) of Braf^cko^ mice begins 2 weeks after birth and is complete at the age of 6 weeks. (**C**) In Western blots from adult mutants, we observed strongly reduced BRAF levels in forebrain regions, a lower reduction in the midbrain, and no reduction in the brainstem and the cerebellum of Braf^cko^ mice. (**D–H**) As shown by immunohistochemistry, the BRAF protein was absent from the glutamatergic neurons of the dentate gyrus (**D, E**), the hippocampus (**F**), and the cortex of mutant mice (**G**). In contrary, BRAF signals in cerebellum (**H**) and in parvalbumin-positive interneurons of the hippocampus were comparable to controls (**I**).
(PDF)

Figure S3 **Behavioral analysis of CamkIIa-Cre mice.** 29 CamkIIa-Cre mice and 29 littermate controls (lacking the Cre transgene) were analyzed and no alterations in locomotion, social interaction, object recognition, anxiety- and depression-related behavior, and motorcoordination were found in the modified hole board (**A–F**), the elevated plus maze (**G**), the forced swim test (**H**), and the accelerating rotarod (**I**). Data of male and female subjects were pooled as no sex differences were found. (n.s.: not significant, *: *P*<0.05).
(PDF)

Figure S4 **GABA_A_ receptor-mediated inhibition is not enhanced in hippocampi from Braf^cko^ mice.** (**A**) Amplitudes of CA1 population spikes (extracellular recording) are plotted as function of stimulus intensity to determine input-output relationship. (**B–D**) are from whole-cell recordings of CA1 pyramidal cells. (**B**) Input-output relationship for evoked IPSCs showed reduced response in CA1 pyramidal cells of Braf^cko^ mice to nearby electrical stimuli delivered close to pyramidal cell layer compared to evoked IPSC responses in control cells (*: p<0.05). (**C**) Train stimuli (delivered at 20 Hz, inset) revealed no significant difference in frequency depression during train between mutant and control neurons. The histogram plots normalized IPSC amplitude relative to amplitude of first IPSC. (**D**) The GABA_A_ receptor agonist muscimol (1 µM) induced similar postsynaptic currents in mutant and control neurons (left columns, p>0.05). Inset illustrates representative muscimol-evoked current response in a control neuron. Downward deflections in the trace reflect spontaneous IPSCs (sp IPSCs), which occurred at about the same frequency in neurons from both lines of mice (right columns, p>0.05).
(PDF)

Table S1 **Gene expression analysis of hippocampal RNA of adult Braf^cko^ mice revealed 165 differentially expressed transcripts (124 downregulated, 41 upregulated,** ***P*** **<0.05).** In total, 150 individual genes were affected, 111 were downregulated and 39 were upregulated. (#: transcripts were found to be also regulated in CamkIIa-Cre controls and therefore excluded from further studies).
(PDF)

Table S2 **qPCR validation of 22 candidate genes from the microarray results.** Genes were classified by gene ontology and MeSH analysis. (*: mean values due to multiple transcripts detected in microarray; s.d.: standard deviation).
(DOC)

Table S3 **Bioinformatic prediction of CREB1 and ETS/SRF target genes.** *: position relative to transcription start site (TSS); #: Hs, Homo sapiens (human); Mm, Mus musculus (mouse); Rn, Rattus norvegicus (rat); Cfa, Canis lupus familiaris (dog); Bt, Bos taurus (cattle); Eca, Equus caballus (horse); Pt, Pan troglodytes (chimpanzee); Mmu Macaca mulatta (rhesus monkey); Ssc, Sus scrofa (wild boar); Gga, Gallus gallus (chicken); Mdm, Monodelphis domestica (short-tailed opossum); Dr, Danio rerio (zebrafish); §: binding sites both on sense and antisense strand; x: sequences aligned in the region of the ETS binding site. All gene symbols, promoter, and transcript identifier were derived from the promoter sequence retrieval database ElDorado (Genomatix, Munich, Germany). Selection of putative target genes was based on significantly downregulated genes from microarray datasets of Braf^cko^ and control mice. Promoter sequences from up to ten different mammalian species were aligned with the DiAlign TF program in the Genomatix software suite GEMS Launcher to evaluate overall promoter similarity and to identify conserved CREB1 and ETS/SRF binding sites (BSs). The promoter regions were defined as ∼900 bp upstream and 100 bp downstream of the transcriptional start site (TSS). Position weight matrices were used according to Matrix Family Library Version 8.1 (June 2009) for promoter analyses. BS or combination of BSs (i.e. Module) were considered as “conserved BS/Module" only if the promoter sequences for all given orthologs could be aligned in the region of CREB1 BS or ETS/SRF Module with the help of the DiAlign TF program (using default settings). The ETS/SRF module was defined by the ModelInspector (Genomatix/GEMS Launcher) with a distance of 9 to 19 bp between the ETS and the SRF BS and was tested for its presence in the c-fos promoter of different species. The genes in the first column were ranked by the degree of conservation of predicted CREB1 BS or ETS/SRF module across ten mammalian species. The higher the amount of conserved sites in an increasing number of orthologous promoter sequences, the higher is the rank of the corresponding gene listed from top to bottom.
(PDF)

Method S1 **Southern blot analysis.**
(PDF)

Method S2 **Behavioral testings: Modified hole board and Accelerating rotarod.**
(PDF)
