# Supplementary material for: MAPK Signaling Determines Anxiety in the Juvenile Mouse Brain but Depression-Like Behavior in Adults
Source: PLoS One. 2012 Apr 18;7(4):e35035. doi: 10.1371/journal.pone.0035035 (PMC3329550; doi:10.1371/journal.pone.0035035)

Figure S1. Structure of the Braf^{flox} allele and expression pattern analysis of the CamkIIa-CreER^{T2} mouse.

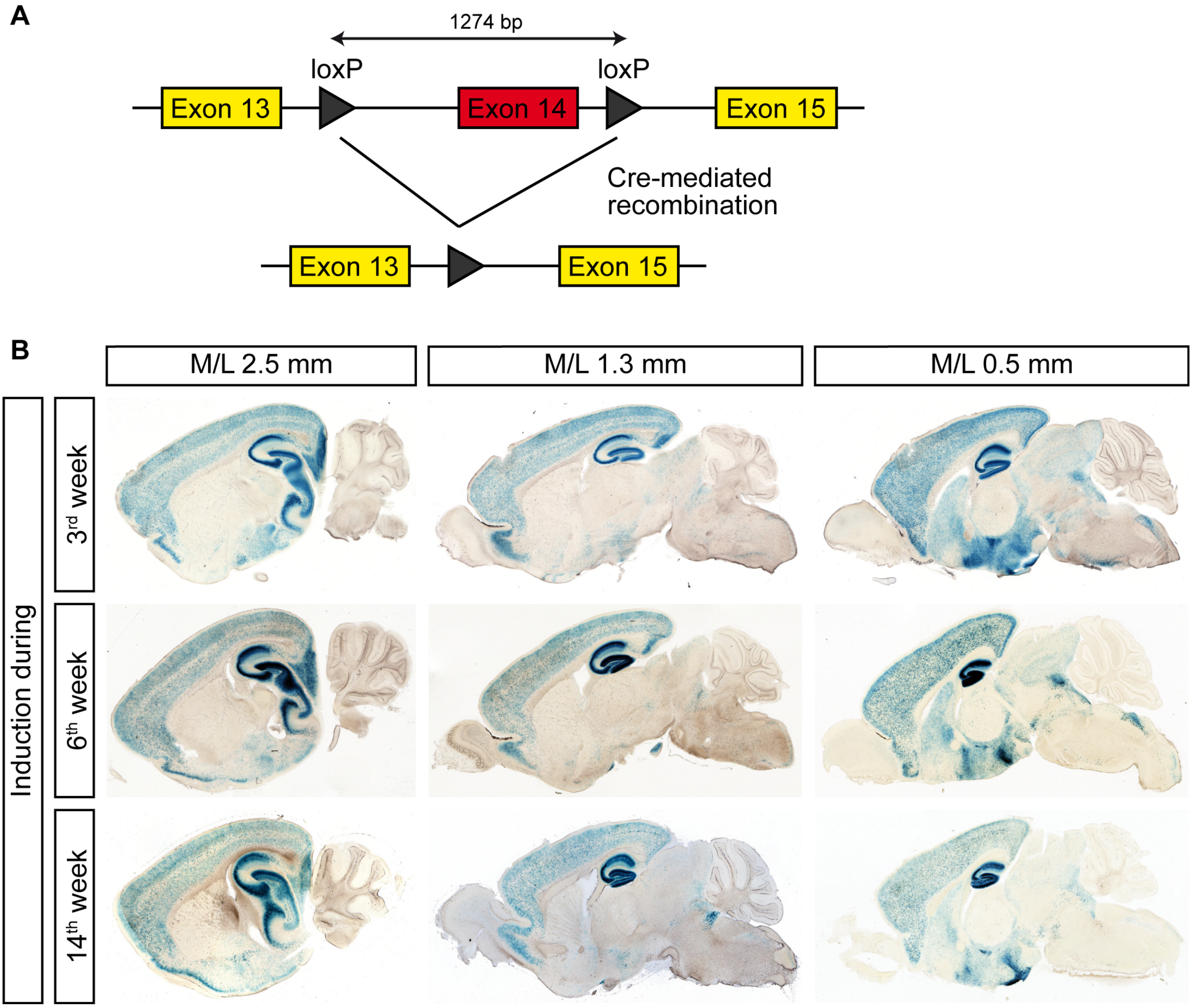

Supplement: Figure S1 — Structure of the Brafflox allele and expression pattern analysis of the CamkIIa-CreERT2 mouse. (A) In the Brafflox allele, exon 14 of Braf is flanked by two Cre recombinase recognition (loxP) sites. Cre mediated excision of this exon leads to a reading frame shift and the production of a truncated, non-functional BRAF protein. (B) CamkIIa-CreERT2 mice were bred with the Gt(ROSA)26Sor Cre reporter line that exhibits β-galactosidase activity upon Cre recombination. Strong recombinase activity was observed in the cortex and hippocampus. Medium activity was found in striatum and hypothalamus. No differences for the induction ability of the CreERT2 were found between the 3rd, 6th, and 14th week of age. (PDF) [file pone.0035035.s001.pdf]
