# Supplementary figures and images for: MAPK Signaling Determines Anxiety in the Juvenile Mouse Brain but Depression-Like Behavior in Adults
Source: PLoS One. 2012 Apr 18;7(4):e35035. doi: 10.1371/journal.pone.0035035 (PMC3329550; doi:10.1371/journal.pone.0035035)

**Figure S2. Molecular characterization of Camklla-Cre and Braf<sup>cko</sup> mice**

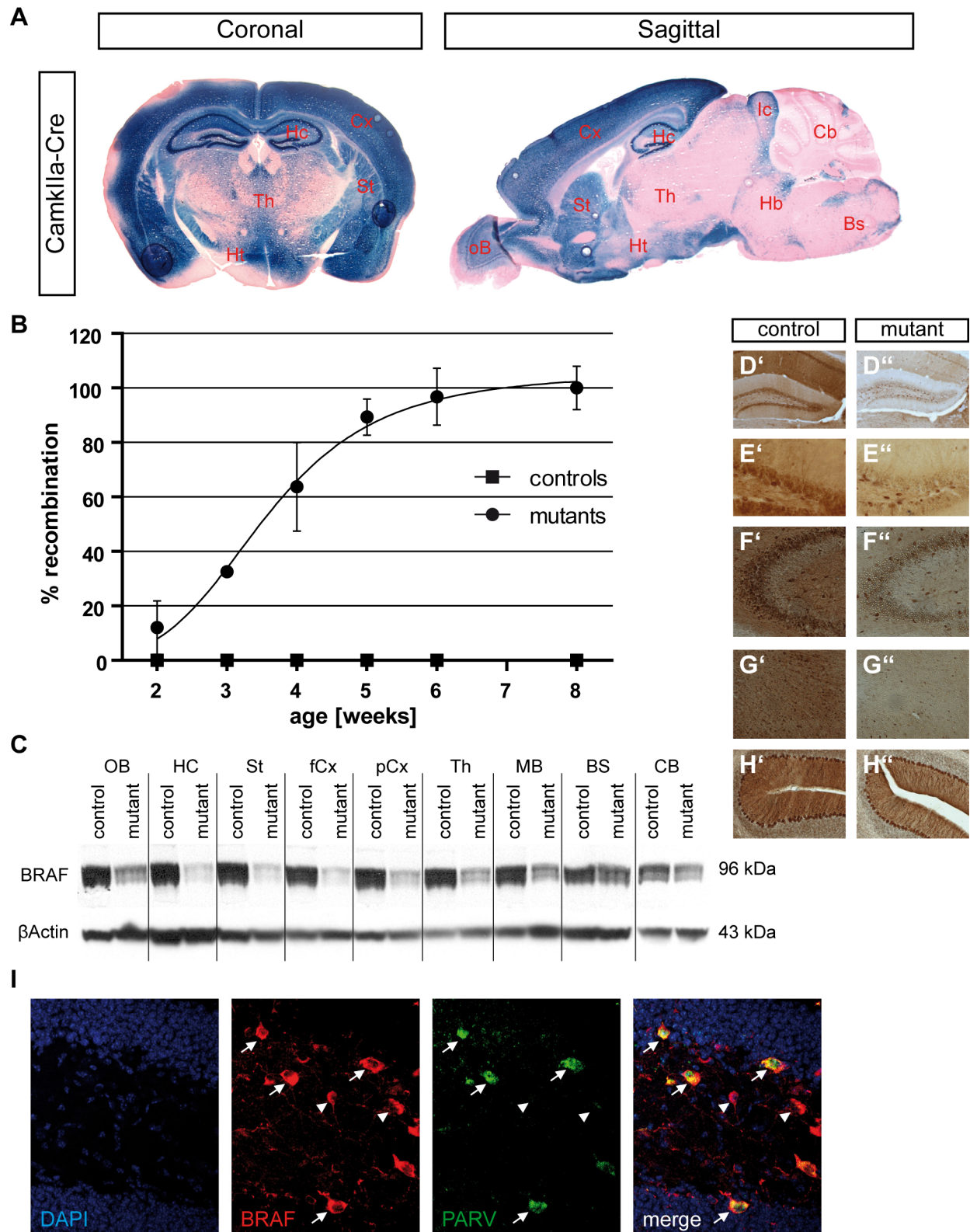

Supplement: Figure S2 — Molecular characterization of CamkIIa-Cre and Brafcko mice. (A) Using a Cre reporter strain (Soriano, 1999), we confirmed the activity of the CamkIIa-Cre transgene in principle forebrain neurons (olfactory bulb (oB), cortex (Cx), hippocampus (Hc), and striatum (St)) and further observed recombination in the hypothalamus (Ht) and inferior colliculus (Ic), but not in the thalamus (Th), hindbrain (Hb), brainstem (Bs), or cerebellum (Cb). (B) Using Southern blot analysis of genomic DNA (Method S1), we found that the inactivation of Braf in the hippocampus and cortex (data not shown) of Brafcko mice begins 2 weeks after birth and is complete at the age of 6 weeks. (C) In Western blots from adult mutants, we observed strongly reduced BRAF levels in forebrain regions, a lower reduction in the midbrain, and no reduction in the brainstem and the cerebellum of Brafcko mice. (D–H) As shown by immunohistochemistry, the BRAF protein was absent from the glutamatergic neurons of the dentate gyrus (D, E), the hippocampus (F), and the cortex of mutant mice (G). In contrary, BRAF signals in cerebellum (H) and in parvalbumin-positive interneurons of the hippocampus were comparable to controls (I). (PDF) [file pone.0035035.s002.pdf]

**Figure S3. Behavioral analysis of Camk1a-Cre mice.**

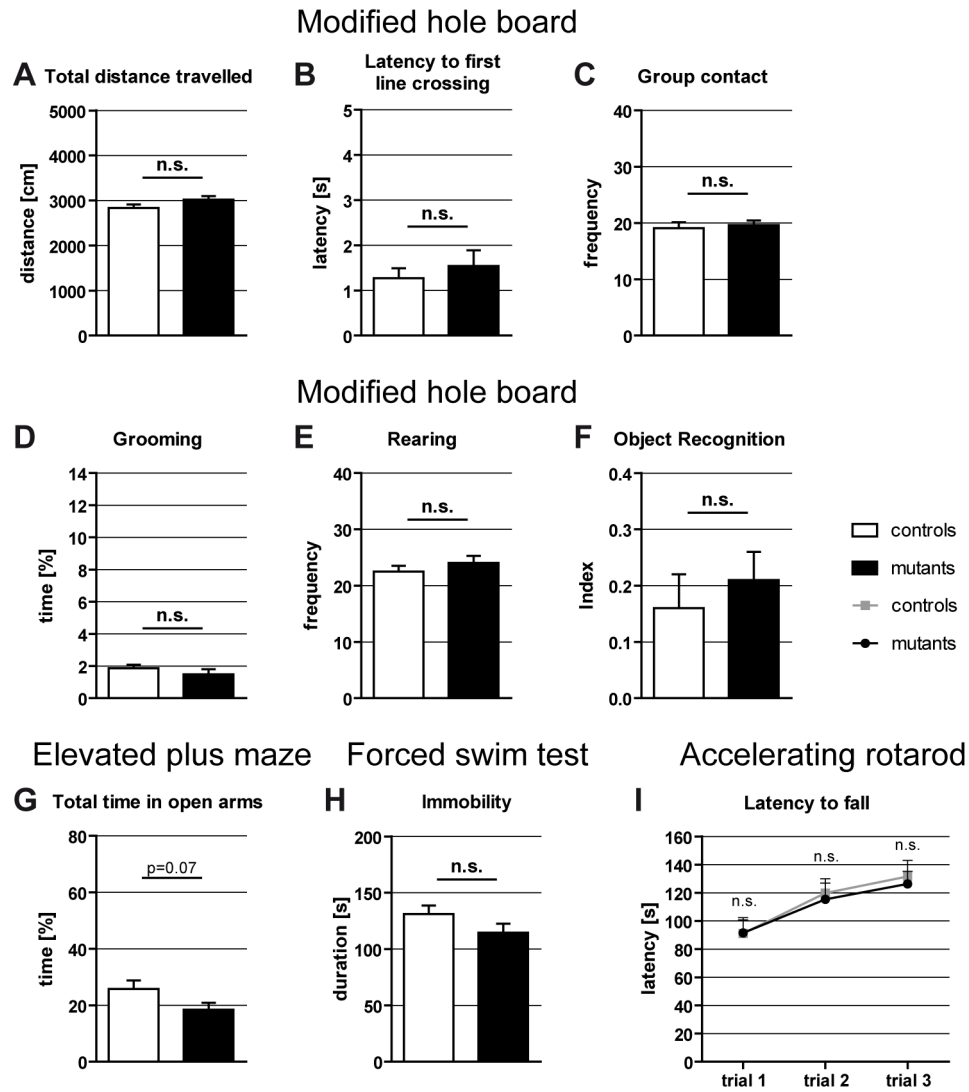

Supplement: Figure S3 — Behavioral analysis of CamkIIa-Cre mice. 29 CamkIIa-Cre mice and 29 littermate controls (lacking the Cre transgene) were analyzed and no alterations in locomotion, social interaction, object recognition, anxiety- and depression-related behavior, and motorcoordination were found in the modified hole board (A–F), the elevated plus maze (G), the forced swim test (H), and the accelerating rotarod (I). Data of male and female subjects were pooled as no sex differences were found. (n.s.: not significant, *: P<0.05). (PDF) [file pone.0035035.s003.pdf]
