# Supplementary material for: MAPK Signaling Determines Anxiety in the Juvenile Mouse Brain but Depression-Like Behavior in Adults
Source: PLoS One. 2012 Apr 18;7(4):e35035. doi: 10.1371/journal.pone.0035035 (PMC3329550; doi:10.1371/journal.pone.0035035)

Figure S4. GABA$_A$ receptor-mediated inhibition is not enhanced in hippocampi from Braf^{cko} mice.

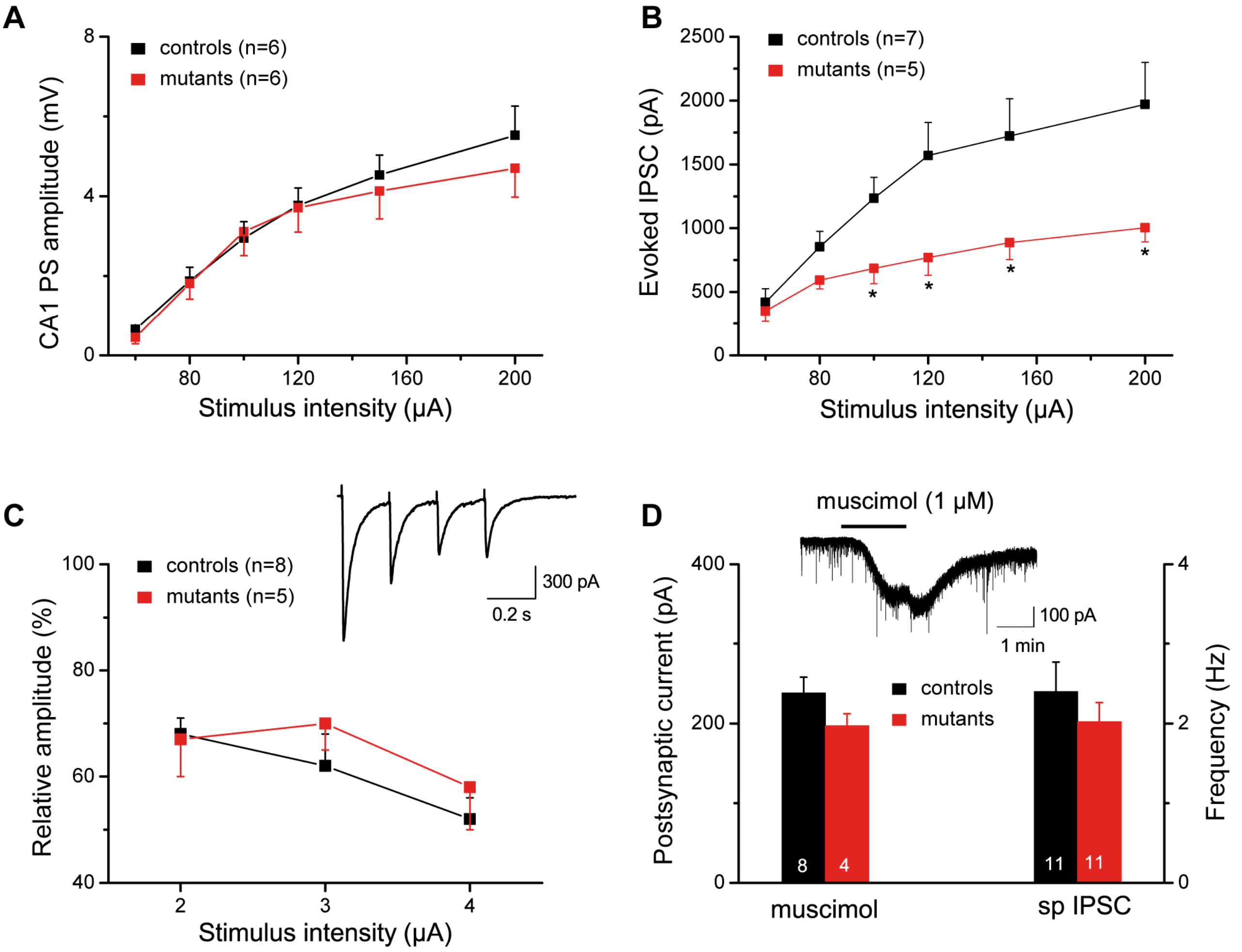

Supplement: Figure S4 — GABAA receptor-mediated inhibition is not enhanced in hippocampi from Brafcko mice. (A) Amplitudes of CA1 population spikes (extracellular recording) are plotted as function of stimulus intensity to determine input-output relationship. (B–D) are from whole-cell recordings of CA1 pyramidal cells. (B) Input-output relationship for evoked IPSCs showed reduced response in CA1 pyramidal cells of Brafcko mice to nearby electrical stimuli delivered close to pyramidal cell layer compared to evoked IPSC responses in control cells (*: p<0.05). (C) Train stimuli (delivered at 20 Hz, inset) revealed no significant difference in frequency depression during train between mutant and control neurons. The histogram plots normalized IPSC amplitude relative to amplitude of first IPSC. (D) The GABAA receptor agonist muscimol (1 µM) induced similar postsynaptic currents in mutant and control neurons (left columns, p>0.05). Inset illustrates representative muscimol-evoked current response in a control neuron. Downward deflections in the trace reflect spontaneous IPSCs (sp IPSCs), which occurred at about the same frequency in neurons from both lines of mice (right columns, p>0.05). (PDF) [file pone.0035035.s004.pdf]
