# Supplementary material for: MAPK Signaling Determines Anxiety in the Juvenile Mouse Brain but Depression-Like Behavior in Adults
Source: PLoS One. 2012 Apr 18;7(4):e35035. doi: 10.1371/journal.pone.0035035 (PMC3329550; doi:10.1371/journal.pone.0035035)
Supplement: Table S1 — Gene expression analysis of hippocampal RNA of adult Brafcko mice revealed 165 differentially expressed transcripts (124 downregulated, 41 upregulated, P <0.05). In total, 150 individual genes were affected, 111 were downregulated and 39 were upregulated. (#: transcripts were found to be also regulated in CamkIIa-Cre controls and therefore excluded from further studies). (PDF) [file pone.0035035.s005.pdf]

Table S1.

Symbol	Name	Accession	fold change	P_{adj}
**downregulated**				
C330006P03Rik	RIKEN cDNA C330006P03 gene	AK049142	-3.04	<0.001
Cyp26b1	cytochrome P450, family 26, subfamily b, polypeptide 1	NM_175475.2	-2.76	<0.001
Dusp6	dual specificity phosphatase 6	NM_026268.1	-2.46	<0.001
Pla2g4e	phospholipase A2, group IVE	NM_177845	-2.46	0.023
Htr5b	5-hydroxytryptamine (serotonin) receptor 5B	NM_010483.2	-2.37	<0.001
Crhbp	corticotropin releasing hormone binding protein	NM_198408.1	-2.35	<0.001
Npy	neuropeptide Y	NM_023456.2	-2.27	<0.001
Egr4	early growth response 4	NM_020596.1	-2.24	0.010
Cort	cortistatin	NM_007745.2	-2.22	0.003
Egr1	early growth response 1	NM_007913.2	-2.13	0.008
Rasd1	RAS, dexamethasone-induced 1	NM_009026.1	-2.11	<0.001
Dusp6	dual specificity phosphatase 6	NM_026268.1	-2.09	0.001
Wnt9a	wingless-type MMTV integration site 9A	NM_139298	-2.05	0.039
Ky	kyphoscoliosis peptidase	NM_024291	-2.03	<0.001
Nptx2	neuronal pentraxin 2	NM_016789.2	-2.01	<0.001
Scg5	secretogranin V	NM_009162.2	-1.97	0.003
Dusp5	dual specificity phosphatase 5	NM_001085390	-1.90	0.021
Oxr1[#]	oxidation resistance 1	NM_130885.1	-1.90	<0.001
Hcrtr1	hypocretin (orexin) receptor 1	NM_198959.1	-1.88	<0.001
Zfpm2[#]	zinc finger protein, multitype 2	NM_011766.2	-1.87	<0.001
Dusp4	dual specificity phosphatase 4	NM_176933	-1.86	0.006
Etv5	ets variant gene 5	NM_023794	-1.85	<0.001
Mboat2	membrane bound O-acyltransferase domain containing 2	NM_026037.2	-1.83	0.023
Efcab6	EF-hand calcium binding domain 6	NM_029946.3	-1.82	<0.001
3110047M12Rik[#]	RIKEN cDNA 3110047M12 gene	AK014186	-1.81	<0.001
Sst	somatostatin	NM_009215.1	-1.80	<0.001
Cort	cortistatin	NM_007745.2	-1.73	0.018
Dusp4	dual specificity phosphatase 4	NM_176933.3	-1.72	0.002
Gpnmb	glycoprotein (transmembrane) nmb	NM_053110.2	-1.72	0.009
Oxr1[#]	oxidation resistance 1	NM_001130166	-1.72	<0.001
Thsd4	thrombospondin, type I, domain containing 4	NM_172444.1	-1.71	0.015
Per2	period homolog 2 (Drosophila)	NM_011066.1	-1.69	0.011
Klk8	kallikrein related-peptidase 8	NM_008940.1	-1.68	0.018
9630021O20Rik	RIKEN clone 9630021O20	AK079321	-1.67	0.040
Synj2	synaptojanin 2	AK038038	-1.66	0.022
C2cd4b	C2 calcium-dependent domain containing 4B	XM_134869.3	-1.66	0.048
Cacna2d1	calcium channel, voltage-dependent, alpha2/delta subunit 1	NM_009784.1	-1.66	0.002
Midn	midnolin	NM_021565.1	-1.64	0.001
Fjx1	four jointed box 1 (Drosophila)	NM_010218.1	-1.62	0.049
Csrnp1	cysteine-serine-rich nuclear protein 1	NM_153287.2	-1.61	0.003
Tnfrsf12a	tumor necrosis factor receptor superfamily, member 12a	NM_013749.1	-1.61	0.009
Zranb3	zinc finger, RAN-binding domain containing 3	NM_027678	-1.60	0.005
2900060B14Rik	RIKEN cDNA 2900060B14 gene	NR_027901	-1.60	0.001
Ryr1	ryanodine receptor 1, skeletal muscle	NM_009109	-1.59	0.006
Tac1	tachykinin 1	NM_009311.1	-1.58	0.006
Trib2	tribbles homolog 2 (Drosophila)	NM_144551.3	-1.58	0.017
LOC329646	hypothetical gene LOC329646	XM_287235.2	-1.56	0.012
Cd59a	CD59a antigen	NM_007652.2	-1.56	0.019
Igfbp4	insulin-like growth factor binding protein 4	NM_010517.2	-1.55	<0.001
Oxr1	oxidation resistance 1	NM_130885.1	-1.55	0.019
Igfbp4	insulin-like growth factor binding protein 4	NM_010517.2	-1.53	0.002
Zbtb7	zinc finger and BTB domain containing 7a	NM_010731.1	-1.53	0.011
Fibcd1	fibrinogen C domain containing 1	NM_178887.2	-1.53	0.034
Masp1	mannan-binding lectin serine peptidase 1	NM_008555	-1.52	0.006
Slc38a5	solute carrier family 38, member 5	NM_172479.1	-1.52	0.003
Camk1g	calcium/calmodulin-dependent protein kinase I gamma	NM_144817.1	-1.52	0.004
2900060N12Rik	RIKEN cDNA 2900060N12 gene	NM_183095.1	-1.51	0.019
Cck	cholecystokinin	NM_031161.1	-1.51	0.003
Car4	carbonic anhydrase 4	NM_007607.1	-1.51	0.034
Chd4	chromodomain helicase DNA binding protein 4	NM_145979.1	-1.51	0.023
Arhgef10	Rho guanine nucleotide exchange factor (GEF) 10	NM_172751.1	-1.50	0.023
Zap70	zeta-chain (TCR) associated protein kinase	NM_009539.2	-1.49	0.011
Zyx	zyxin	NM_011777.1	-1.48	0.008
Shc3	src homology 2 domain-containing transforming protein C3	NM_009167.1	-1.48	0.040
Gm336	predicted gene 336	XM_140607.1	-1.47	0.015
Etv1	ets variant gene 1	NM_007960.1	-1.46	0.012

Mtap2	microtubule-associated protein 2	AK079618	-1.46	0.037
Spred1	sprouty protein with EVH-1 domain 1, related sequence	NM_033524	-1.45	0.004
Calb1	calbindin 1	NM_009788	-1.45	0.023
Pde1a	phosphodiesterase 1A, calmodulin-dependent	NM_016744.1	-1.44	0.034
Tpd52l1	tumor protein D52-like 1	NM_009413.1	-1.44	0.009
Bcl6	B-cell leukemia/lymphoma 6	NM_009744.2	-1.44	0.023
Hs3st1	heparan sulfate (glucosamine) 3-O-sulfotransferase 1	NM_010474.1	-1.43	0.006
A630022G20Rik	RIKEN clone A630022G20	AK041581	-1.43	0.008
Ppapdc2	phosphatidic acid phosphatase type 2 domain containing 2	NM_028922	-1.42	0.010
Fam19a1	family with sequence similarity 19, member A1	NM_182808.1	-1.42	0.010
Pex5l	peroxisomal biogenesis factor 5-like	AK044552.1	-1.42	0.020
Asap2	ArfGAP with SH3 domain, ankyrin repeat and PH domain 2	NM_001004364	-1.42	0.014
A130009M03Rik	RIKEN clone A130009M03	AK037351	-1.42	0.033
Osbpl3	oxysterol binding protein-like 3	NM_027881.1	-1.41	0.025
Bdnf	brain derived neurotrophic factor	AY057913	-1.41	0.036
Cdh10	cadherin 10	NM_009865	-1.41	0.009
Camk1g	calcium/calmodulin-dependent protein kinase I gamma	NM_144817	-1.40	0.019
Bach2	BTB and CNC homology 2	NM_007521.2	-1.40	0.011
Tpd52l1	tumor protein D52-like 1	NM_009413.1	-1.39	0.016
Etv1	ets variant gene 1	NM_007960.1	-1.38	0.033
Zfpm1	zinc finger protein, multitype 1	NM_009569.1	-1.38	0.019
A630020C08Rik	RIKEN clone A630020C08	AK041540	-1.37	0.022
Tuft1	tuftelin 1	NM_011656.1	-1.37	0.023
Ttc39b	tetratricopeptide repeat domain 39B	NM_025782.2	-1.37	0.048
Fam19a1	family with sequence similarity 19, member A1	NM_182808	-1.36	0.022
Stard8	START domain containing 8	NM_199018.1	-1.36	0.045
Zfp326	zinc finger protein 326	NM_018759.1	-1.36	0.034
Scn3b	sodium channel, voltage-gated, type III, beta	NM_153522.1	-1.36	0.019
Neto2	neuropilin (NRP) and tolloid (TLL)-like 2	NM_001081324	-1.35	0.019
C030027H14Rik	RIKEN cDNA C030027H14 gene	AK021106	-1.35	0.042
Rhobtb1	Rho-related BTB domain containing 1	XM_125637	-1.34	0.039
Sgk1	serum/glucocorticoid regulated kinase 1	NM_011361	-1.34	0.042
Gria3	glutamate receptor, ionotropic, AMPA3 (alpha 3)	NM_016886.1	-1.34	0.027
Itpr1	inositol 1,4,5-triphosphate receptor 1	NM_010585.2	-1.34	0.021
Sept6	septin 6	NM_019942.2	-1.34	0.019
Homer1	homer homolog 1 (Drosophila)	NM_147176.1	-1.34	0.049
Anks1b	ankyrin repeat and sterile alpha motif domain containing 1B	NM_181398.2	-1.33	0.049
Pramef8	PRAME family member 8	NM_172877.1	-1.33	0.016
Lmo2	LIM domain only 2	NM_008505.3	-1.32	0.049
Fam98c	family with sequence similarity 98, member C	NM_028661.1	-1.32	0.044
Bhlhe40	basic helix-loop-helix family, member e40	NM_011498.2	-1.31	0.017
Tns1	tensin 1	XM_355214.1	-1.31	0.027
Mast4	microtubule associated serine/threonine kinase family member 4	XM_283179.2	-1.31	0.042
Exph5	exophilin 5	NM_176846	-1.31	0.030
Elmo2	engulfment and cell motility 2, ced-12 homolog (C. elegans)	NM_080287.2	-1.31	0.042
Magi1	membrane associated guanylate kinase, WW and PDZ domain containing 1	AK031353	-1.31	0.042
Dcbld1	discoidin, CUB and LCCL domain containing 1	NM_025705	-1.31	0.034
Cds1	CDP-diacylglycerol synthase 1	NM_173370.3	-1.30	0.048
Tmem25	transmembrane protein 25	NM_027865.1	-1.30	0.038
Lypla2	lysophospholipase 2	NM_011942.1	-1.30	0.026
C030007I01Rik	RIKEN cDNA C030007I01 gene	AK021055	-1.30	0.036
9330154F10Rik	RIKEN cDNA 9330154F10 gene	AK020373	-1.30	0.034
D15Wsu169e	DNA segment, Chr 15, Wayne State University 169, expressed	NM_198420.1	-1.29	0.031
A830055N07Rik	RIKEN cDNA A830055N07 gene	AK034366	-1.29	0.048
Trappc6b	trafficking protein particle complex 6B	XM_127025.2	-1.28	0.049
Rlbp1	retinaldehyde binding protein 1	NM_020599.1	-1.28	0.048
Psmd8	proteasome (prosome, macropain) 26S subunit, non-ATPase, 8	NM_026545.1	-1.27	0.049
LOC383514	similar to Lix1 protein (LOC383514)	XM_357101.1	-1.27	0.048
**upregulated**				
Oprl1	opioid receptor-like 1	NM_011012.2	1.26	0.048
Dgat2	diacylglycerol O-acyltransferase 2	NM_026384.2	1.27	0.049
Magi3	membrane associated guanylate kinase, WW and PDZ domain containing 3	NM_133853	1.29	0.040
Syn2	synapsin II	AK043584	1.30	0.047
D530007H06Rik	RIKEN clone D530007H06	AK052561	1.30	0.042
Mycl1	v-myc myelocytomatosis viral oncogene homolog 1, lung carcinoma derived (avian)	NM_008506.2	1.31	0.030
Pak4	p21 protein (Cdc42/Rac)-activated kinase 4	NM_027470.2	1.32	0.022
Cdca7l	cell division cycle associated 7 like	NM_146040.1	1.33	0.019
9530019H20Rik	RIKEN cDNA 9530019H20 gene	NM_177308.2	1.33	0.047
Mbd4	methyl-CpG binding domain protein 4	NM_010774.1	1.33	0.033
Ehd1	EH-domain containing 1	NM_010119.3	1.34	0.016

Cygb	cytoglobin	NM_030206.1	1.34	0.017
C630001G18Rik	RIKEN cDNA C630001G18 gene	AK048185	1.35	0.013
D230046F09Rik	RIKEN clone D230046F09	AK052106	1.38	0.022
Neurod2	neurogenic differentiation 2	NM_010895	1.39	0.023
Lypd1	Ly6/Plaur domain containing 1	NM_145100.2	1.39	0.035
Cxcl12	chemokine (C-X-C motif) ligand 12	NM_021704.1	1.40	0.049
Cacna1g	calcium channel, voltage-dependent, T type, alpha 1G subunit	NM_009783.1	1.41	0.026
Abcb10	ATP-binding cassette, sub-family B (MDR/TAP), member 10	NM_019552.1	1.41	0.034
Slc24a4	solute carrier family 24 (sodium/potassium/calcium exchanger), member 4	NM_172152	1.41	0.014
C030009J22Rik	RIKEN cDNA C030009J22 gene	AK044617	1.41	0.019
Ncrna00081	non-protein coding RNA 81	NR_027828	1.41	0.022
Igfbp5	insulin-like growth factor binding protein 5	NM_010518	1.43	0.016
2900084I15Rik	RIKEN cDNA 2900084I15 gene	AK013819	1.43	0.006
Prss12	protease, serine, 12 neurotrypsin (motopsin)	NM_008939.1	1.43	0.022
Smoc2	SPARC related modular calcium binding 2	NM_022315.1	1.48	0.019
Ntng2	netrin G2	NM_133500	1.48	0.002
Spata13	spermatogenesis associated 13	XM_147847.4	1.50	0.009
B930095M22Rik	RIKEN cDNA B930095M22 gene	AK047590	1.50	0.006
Lpl	lipoprotein lipase	NM_008509.1	1.50	0.041
Slc24a4	solute carrier family 24 (sodium/potassium/calcium exchanger), member 4	NM_172152.1	1.54	0.003
Gpc3	glypican 3	NM_016697.2	1.54	0.002
Fam101b	family with sequence similarity 101, member B	XM_203453	1.54	0.023
Prss12	protease, serine, 12 neurotrypsin (motopsin)	NM_008939.1	1.54	0.006
Nos1	nitric oxide synthase 1, neuronal	NM_008712	1.59	0.013
Gpr68	G protein-coupled receptor 68	NM_175493.2	1.63	0.047
Wnk4	WNK lysine deficient protein kinase 4	NM_175638	1.67	0.005
Rasl10a	RAS-like, family 10, member A	NM_145216.2	1.73	0.002
Cartpt	CART prepropeptide	NM_013732.3	1.75	<0.001
Rnf170	ring finger protein 170	NM_029965	1.81	<0.001
Ctdspl2	CTD small phosphatase like 2	NM_212450	1.99	0.026
