# Supplementary material for: MAPK Signaling Determines Anxiety in the Juvenile Mouse Brain but Depression-Like Behavior in Adults
Source: PLoS One. 2012 Apr 18;7(4):e35035. doi: 10.1371/journal.pone.0035035 (PMC3329550; doi:10.1371/journal.pone.0035035)
Supplement: Table S2 — qPCR validation of 22 candidate genes from the microarray results. Genes were classified by gene ontology and MeSH analysis. (*: mean values due to multiple transcripts detected in microarray; s.d.: standard deviation). (DOC) [file pone.0035035.s006.doc]

Table S2.

|  |  | **Microarray** | |  | **qPCR Validation** | |  | **Transcription factor** | **IEG** | **Behavior-related** | **Neuronal development** | **Circadian Rhythm** |
| --- | --- | --- | --- | --- | --- | --- | --- | --- | --- | --- | --- | --- |
| **Symbol** | **Gene name** | **ratio** | ***P_adj_*** |  | **ratio** | **s.d.** |  |  |  |  |  |  |
| Bach2 | BTB and CNC homology 2 | 0.71 | <0.02 |  | 0.75 | 0.14 |  | x |  |  |  |  |
| Bcl6 | B-cell leukemia/lymphoma 6 | 0.69 | <0.05 |  | 0.72 | 0.18 |  | x |  |  |  |  |
| Bdnf | brain derived neurotrophic factor | 0.71 | <0.05 |  | 0.44 | 0.08 |  |  | x | x | x | x |
| Bhlhe40 | basic helix-loop-helix family, member e40 | 0.76 | <0.02 |  | 0.65 | 0.06 |  | x |  | x |  | x |
| Camk1g | calcium/calmodulin-dep. protein kinase I gamma | 0.68* | <0.02* |  | 0.57 | 0.10 |  |  | x | x |  |  |
| Cck | cholecystokinin | 0.66 | <0.01 |  | 0.61 | 0.08 |  |  | x | x | x | x |
| Crhbp | corticotropin releasing hormone binding protein | 0.43 | <0.001 |  | 0.42 | 0.10 |  |  |  | x |  |  |
| Dusp6 | dual specificity phosphatase 6 | 0.44* | <0.01* |  | 0.25 | 0.06 |  |  | x |  |  | x |
| Egr1 | early growth response 1, Zif268 | 0.47 | <0.01 |  | 0.28 | 0.03 |  | x | x | x | x | x |
| Egr4 | early growth response 4 | 0.45 | <0.01 |  | 0.34 | 0.08 |  | x | x |  |  |  |
| Etv1 | ets variant gene 1 | 0.70* | <0.05* |  | 0.57 | 0.07 |  | x |  | x |  |  |
| Etv5 | ets variant gene 5 | 0.54 | <0.001 |  | 0.44 | 0.06 |  | x |  |  |  |  |
| Gria3 | glutamate receptor, ionotropic, AMPA3 (alpha 3) | 0.75 | <0.05 |  | 0.88 | 0.10 |  |  |  | x |  |  |
| Homer1 | homer homolog 1 (Drosophila) | 0.75 | <0.05 |  | 0.49 | 0.05 |  |  |  | x |  |  |
| Klk8 | kallikrein related-peptidase 8 | 0.60 | <0.02 |  | 0.52 | 0.07 |  |  |  | x | x |  |
| Mycl1 | v-myc myelocytomatosis viral oncogene homolog 1 | 1.31 | <0.05 |  | 1.32 | 0.19 |  | x |  |  |  |  |
| Npy | Neuropeptide Y | 0.44 | <0.001 |  | 0.40 | 0.05 |  |  | x | x |  |  |
| Per2 | period homolog 2 (Drosophila) | 0.59 | <0.02 |  | 0.52 | 0.08 |  | x |  | x |  | x |
| Prss12 | protease, serine, 12 neurotrypsin (motopsin) | 1.49* | <0.02* |  | 1.53 | 0.13 |  |  |  | x |  |  |
| Rasd1 | RAS, dexamethasone-induced 1 | 0.47 | <0.001 |  | 0.28 | 0.10 |  |  |  |  |  | x |
| Spred1 | sprouty protein with EVH-1 domain 1, rel. sequence | 0.69 | <0.01 |  | 0.52 | 0.04 |  |  |  | x |  |  |
| Zfpm1 | zinc finger protein, multitype 1 | 0.73 | <0.02 |  | 0.65 | 0.14 |  | x |  |  |  |  |
