# Supplementary material for: MAPK Signaling Determines Anxiety in the Juvenile Mouse Brain but Depression-Like Behavior in Adults
Source: PLoS One. 2012 Apr 18;7(4):e35035. doi: 10.1371/journal.pone.0035035 (PMC3329550; doi:10.1371/journal.pone.0035035)
Supplement: Table S3 — Bioinformatic prediction of CREB1 and ETS/SRF target genes. *: position relative to transcription start site (TSS); #: Hs, Homo sapiens (human); Mm, Mus musculus (mouse); Rn, Rattus norvegicus (rat); Cfa, Canis lupus familiaris (dog); Bt, Bos taurus (cattle); Eca, Equus caballus (horse); Pt, Pan troglodytes (chimpanzee); Mmu Macaca mulatta (rhesus monkey); Ssc, Sus scrofa (wild boar); Gga, Gallus gallus (chicken); Mdm, Monodelphis domestica (short-tailed opossum); Dr, Danio rerio (zebrafish); §: binding sites both on sense and antisense strand; x: sequences aligned in the region of the ETS binding site. All gene symbols, promoter, and transcript identifier were derived from the promoter sequence retrieval database ElDorado (Genomatix, Munich, Germany). Selection of putative target genes was based on significantly downregulated genes from microarray datasets of Brafcko and control mice. Promoter sequences from up to ten different mammalian species were aligned with the DiAlign TF program in the Genomatix software suite GEMS Launcher to evaluate overall promoter similarity and to identify conserved CREB1 and ETS/SRF binding sites (BSs). The promoter regions were defined as ∼900 bp upstream and 100 bp downstream of the transcriptional start site (TSS). Position weight matrices were used according to Matrix Family Library Version 8.1 (June 2009) for promoter analyses. BS or combination of BSs (i.e. Module) were considered as “conserved BS/Module" only if the promoter sequences for all given orthologs could be aligned in the region of CREB1 BS or ETS/SRF Module with the help of the DiAlign TF program (using default settings). The ETS/SRF module was defined by the ModelInspector (Genomatix/GEMS Launcher) with a distance of 9 to 19 bp between the ETS and the SRF BS and was tested for its presence in the c-fos promoter of different species. The genes in the first column were ranked by the degree of conservation of predicted CREB1 BS or ETS/SRF module across ten mammalian sp [file pone.0035035.s007.pdf]

Table S3. Bioinformatic prediction of CREB1 and ETS/SRF target genes.

			Promoter sequences with BS					
Gene symbol	Binding site (BS)	BS in mouse promoter location*	# of species	Ortholog[#]	Conserved BS / Module	fold change in microarray	Promoter ID	Transcript ID
Sst	CREB1	−55 bp	10	Hs, Mm, Rn, Bt, Cfa, Pt, Mmu, Ssc, Mdm, Dr	2[§]	−1.80	GXP_82140	NM_009215
Gria3	CREB1	−885 bp	8	Hs, Mm, Bt, Cfa, Eca, Pt, Mmu, Mdm	1	−1.34	GXP_229843	AK046158
Dusp4	CREB1	−459 bp	8	Hs, Mm, Rn, Cfa, Eca, Pt, Mmu, Dr	1	−1.86	GXP_270699	NM_176933
	ETS/SRF	+17 bp	7	Hs, Mm, Rn, Cfa, Eca, Pt, Mmu	1			
Egr1	CREB1	−145 to −76 bp	6	Hs, Mm, Bt, Cfa, Gga, Dr	2	−2.13	GXP_423205	NM_007913
	ETS/SRF	−422 to −82 bp	4	Hs, Mm, Bt, Cfa	4			
D15Wsu169e	CREB1	−74 bp	6	Hs, Mm, Rn, Bt, Pt, Mmu	1	−1.29	GXP_295561	AK035479
	ETS/SRF	−96 bp	2	Mm, Rn	1			
Zfp326	ETS	−113 bp	5	Hs, Mm, Bt, Pt, Mmu	1	−1.36	GXP_152119	AK017693
Bdnf (transcript variant 3)	CREB1	−45 bp	4	Hs, Mm, Eca, Pt	1	−1.41	GXP_892129	NM_001048141
Dusp5	CREB1	−238 bp	3	Mm, Bt, Ssc	1	−1.90	GXP_313715	NM_001085390
	ETS/SRF	−271 bp	4	Hs, Mm, Bt, Ssc	1			
Egr4	CREB1	−234 to −166 bp	3	Mm, Bt, Pt	2	−2.24	GXP_303501	NM_020596
