# Supplementary material for: MAPK Signaling Determines Anxiety in the Juvenile Mouse Brain but Depression-Like Behavior in Adults
Source: PLoS One. 2012 Apr 18;7(4):e35035. doi: 10.1371/journal.pone.0035035 (PMC3329550; doi:10.1371/journal.pone.0035035)
Supplement: Method S1 — Southern blot analysis. (PDF) [file pone.0035035.s008.pdf]

Supporting Method S1

Southern blot analysis

Genomic DNA was isolated from tissue samples with phenol/chloroform extraction, digested with HindIII, separated by gel electrophoresis, and transferred to Hybond N+ membranes. A 440 bp genomic fragment, ranging from intron 12 to 13 of the *Braf* gene, was amplified using PCR (forward primer: 5'-AGG CAC AGG AAC TTG GGA GT-3', reverse primer: 5'-TCC GAG GAT GAG GAA GAA GA-3') and used as P^{32}-labeled probe to detect the $Braf^{flox}$ and the recombined alleles. The membranes were next exposed to Kodak X-ray films and signals were quantified with ImageJ.
