# Supplementary material for: MAPK Signaling Determines Anxiety in the Juvenile Mouse Brain but Depression-Like Behavior in Adults
Source: PLoS One. 2012 Apr 18;7(4):e35035. doi: 10.1371/journal.pone.0035035 (PMC3329550; doi:10.1371/journal.pone.0035035)
Supplement: Method S2 — Behavioral testings: Modified hole board and Accelerating rotarod. (PDF) [file pone.0035035.s009.pdf]

Supporting Method S2

Behavioral testings

Modified hole board:

The modified hole board test was carried out as previously described [1]. The test apparatus consisted of a box (150 x 50 x 50 cm) that was divided into a test arena (100 x 50 cm) and a group compartment (50 x 50 cm) by a transparent PVC partition (50 x 50 x 0.5 cm). Both box and board were made of dark grey PVC. The illumination levels were set at approximately 150 lux in the corners and 200 lux in the middle of the test arena. At the beginning of the experiment, all animals of a cage were habituated to the test environment in the group compartment for 20 min. Then each animal was placed individually into the test arena and allowed to explore it freely for 5 min, while the cage mates remained in the group compartment. Animals were always placed in the same corner of the test arena next to the partition, facing the board diagonally. During the 5 min trial, the animal's behavior was recorded by a trained observer with a hand-held computer. Data were analyzed by using the Observer 4.1 Software (Noldus, Wageningen, Netherlands). Additionally, a camera was mounted 1.20 m above the centre of the test arena, and the animal's track was videotaped and its locomotor path analyzed with a video-tracking system (Ethovision 2.3, Noldus). After each trial, the test arena was cleaned carefully with a disinfectant.

Accelerating rotarod:

Motor coordination and balance were assessed using the rotating rod apparatus from Bioseb (Letica LE 8200). The rod diameter was approx. 4.5 cm made of hard plastic material covered by soft black rubber foam with a lane width of approx. 5 cm. The test phase consisted of three trials separated by 15 min intertrial intervals. On each trial, three mice were placed on the rod leaving an empty lane between two mice. The rod was initially rotating at 4 rpm constant speed to allow positioning of all mice in their respective lanes. Once all mice were positioned, the trial was started and the rod accelerated from 4 rpm to 40 rpm in 300 sec. Latency and rpm at which each mouse fell off the rod were measured. Passive rotations were counted as a fall and the mouse was immediately carefully removed from the rod. After each trial, the apparatus was disinfected and let dry. This protocol is based on the EUMODIC EMPReSS Slim standard operating procedure (see www.eumodic.org).

References

1. Vauti F, Goller T, Beine R, Becker L, Klopstock T, et al. (2007) The mouse Trm1-like gene is expressed in neural tissues and plays a role in motor coordination and exploratory behaviour. Gene 389: 174-185.
